# A genome-wide association study of tinnitus reveals shared genetic links to neuropsychiatric disorders

**DOI:** 10.1038/s41598-022-26413-6

**Published:** 2022-12-29

**Authors:** Ishan Sunilkumar Bhatt, Nicholas Wilson, Raquel Dias, Ali Torkamani

**Affiliations:** 1 Department of Communication Sciences & Disorders, University of Iowa, 250 Hawkins Dr, Iowa City, IA 52242 USA; 2 Department of Integrative Structural and Computational Biology Scripps Science Institute, La Jolla, CA 92037 USA; 3 Department of Microbiology and Cell Science, University of Florida, Gainesville, FL 32608 USA

**Keywords:** Genome-wide association studies, Risk factors

## Abstract

Tinnitus, a phantom perception of sound in the absence of any external sound source, is a prevalent health condition often accompanied by psychiatric comorbidities. Recent genome-wide association studies (GWAS) highlighted a polygenic nature of tinnitus susceptibility. A shared genetic component between tinnitus and psychiatric conditions remains elusive. Here we present a GWAS using the UK Biobank to investigate the genetic processes linked to tinnitus and tinnitus-related distress, followed by gene-set enrichment analyses. The UK Biobank sample comprised 132,438 individuals with tinnitus and genotype data. Among the study sample, 38,525 individuals reported tinnitus, and 26,889 participants mentioned they experienced tinnitus-related distress in daily living. The genome-wide association analyses were conducted on tinnitus and tinnitus-related distress. We conducted enrichment analyses using FUMA to further understand the genetic processes linked to tinnitus and tinnitus-related distress. A genome-wide significant locus (lead SNP: rs71595470) for tinnitus was obtained in the vicinity of *GPM6A*. Nineteen independent loci reached suggestive association with tinnitus. Fifteen independent loci reached suggestive association with tinnitus-related distress. The enrichment analysis revealed a shared genetic component between tinnitus and psychiatric traits, such as bipolar disorder, feeling worried, cognitive ability, fast beta electroencephalogram, and sensation seeking. Metabolic, cardiovascular, hematological, and pharmacological gene sets revealed a significant association with tinnitus. Anxiety and stress-related gene sets revealed a significant association with tinnitus-related distress. The GWAS signals for tinnitus were enriched in the hippocampus and cortex, and for tinnitus-related distress were enriched in the brain and spinal cord. This study provides novel insights into genetic processes associated with tinnitus and tinnitus-related distress and demonstrates a shared genetic component underlying tinnitus and psychiatric conditions. Further collaborative attempts are necessary to identify genetic components underlying the phenotypic heterogeneity in tinnitus and provide biological insight into the etiology.

## Introduction

Tinnitus is a phantom perception of sound in the absence of any external sound source. About 21 million US adults have experienced bothersome tinnitus, with 27% of tinnitus sufferers reporting symptoms for longer than 15 years^1^. About 2 million US adults experience an extreme form of debilitating tinnitus^2^. Individuals with debilitating tinnitus experience a severe manifestation of tinnitus-related distress in daily living, often characterized by chronic insomnia, inability to concentrate and relax, persistent anxiety and depression, and severe hyperacusis (heightened reaction to routine sounds)^3^. Tinnitus is a common hearing health concern among individuals exposed to intense noise^4,5^. About 15% of workers exposed to occupational noise experience bothersome tinnitus. Nearly 40% of workers with tinnitus believe that their tinnitus adversely affects their work, and 25% do not prefer to discuss their problems with others due to fear that it might affect their employment opportunities^5^. Personal economic loss to an individual with tinnitus can be up to $30,000/year, and the burden to society can be $26 billion/year^3^.

Tinnitus is a common hearing health concern in individuals with hearing loss (e.g.,^6^). Past studies suggest cochlear deafferentation can play a role in triggering tinnitus perception^7^. While peripheral cochlear damage and maladaptive central compensation are associated with tinnitus genesis, tinnitus-related distress is associated with altered cortical networks^8^. Several non-auditory structures such as the amygdala, hippocampus, middle and superior frontal gyri, cingulate gyrus, precuneus, and the parietal cortices are also associated with tinnitus^9^. Systematic diseases, such as hypertension and diabetes, can affect tinnitus perception (e.g.,^10^). Psychological factors, such as stress, anxiety, and depression, can also modify tinnitus perception^11^. Collectively, this multi-layered pathological mechanism can influence the phenotypic spectrum of tinnitus.

Recreational and occupational noise exposure is associated with tinnitus in large-scale epidemiological studies^1–4,12^. Smoking, ototoxic medications, and chemical exposure are significant risk factors for tinnitus^1–4,13^. Cardiovascular conditions such as hypertension, heart diseases, stroke, ischemia, and arterial hypertension are associated with tinnitus^14^. Common medical drugs for treating cardiovascular conditions, such as diuretics, angiotensin-converting enzyme inhibitors, and calcium channel blockers, are associated with tinnitus^15^. Tinnitus is often accompanied by psychiatric conditions such as anxiety, depression, and bipolar disorder^16^. A Mendelian randomization study using the UK Biobank database identified hearing loss, major depression, neuroticism, and high systolic blood pressure associated with tinnitus^17^.

The genetic interrogation of tinnitus can help identify the underlying molecular mechanisms. Twin studies suggested a genetic component to tinnitus susceptibility. Tinnitus heritability (h2) ranges from 0.21 to 0.68^18–21^. The heritability estimate is about 0.68 for bilateral tinnitus in men and 0.41 in females^21^. Past genetic studies investigating tinnitus in a candidate gene set had limited success in identifying novel genetic variants involved in tinnitus (e.g.,^22^). Recent genome-wide association studies (GWAS) are beginning to illuminate the polygenic nature of tinnitus susceptibility^23–27^. Two previous GWAS used the UK Biobank database to investigate the genetic variants underlying tinnitus. Clifford et al. identified six genome-wide significant loci associated with tinnitus in the UK Biobank cohort and replicated three of them in the Million Veteran Program cohort^23^. A recent study using the case–control approach with the UK Biobank database identified three variants in the vicinity of *RCOR1* gene^24^. Here we conduct a complimentary GWAS analysis in the UK Biobank, adjusting for known environmental risk factors, and interrogating the genetic underpinnings of tinnitus-related distress. In addition, we conduct enrichment analyses to further understand the genetic processes linked to tinnitus and tinnitus-related distress. We find that gene sets specifically expressed in the central nervous system and underpinning neuropsychiatric disorders are enriched in GWAS significant loci for both tinnitus and tinnitus-related distress.

## Methods

The ethical approval for the present study was obtained from the University of Iowa Institutional Review Board. The UK Biobank approved the research application, and the research was conducted per the UK Biobank regulations and guidelines. The informed consent was taken from all study participants. The UK Biobank database containing the demographic variables, questionnaire responses, and genome-wide single nucleotide polymorphism (SNP) markers were obtained (Project ID: 68779). The database includes data from > 500,000 participants assessed across the UK from 2006 to 2010. The volunteers donated blood samples while visiting a UK Biobank assessment center. The complete details of the blood sample collection and DNA extraction process are described earlier^28^.

### Tinnitus phenotype and questionnaire responses

The participants filled out a touchscreen questionnaire at the UK Biobank assessment center. The question (Data-filed #4803) text was, “Do you get or have you had noises (such as ringing or buzzing) in your head or in one or both ears that last for more than five minutes at a time?”. The answer choice included, “Yes, now most or all of the time”, “Yes, now a lot of the time”, “Yes, now some of the time”, “Yes, but not now, but had in the past”, “No, never”, “Do not know”, and “Prefer not to answer”. We removed individuals answering, “Do not know” and “Prefer not to answer” from the study sample and created an ordinal variable with four levels—“No, never”, “Yes, but not now”, “Yes, now some of the time”, “Yes, now a lot of the time”.

Individuals reporting tinnitus were further questioned about tinnitus-related distress with (Data-filed #4814), “How much do these noises worry, annoy, or upset you when they are at their worst?”. The answer choices included “Severely”, “Moderately”, “Slightly”, “Not at all”, “Do not know”, and “Prefer not to answer”. We removed individuals answering, “Do not know” and “Prefer not to answer” from the study sample. We created an ordinal variable of five levels—“No, never (from Data-filed #4803)”, “Not at all”, “Slightly”, “Moderately”, and “Severely”. We reasoned individuals with no tinnitus would not experience tinnitus-related distress.

The questionnaire responses were used for evaluating potential confounders and covariates. The demographic variables such as age, sex, and ethnicity were extracted from the database. Occupational noise exposure was investigated by (Data-field #4825), “Have you ever worked in a noisy place where you had to shout to be heard?”. Recreational noise exposure was evaluated by (Data-field #4836), “Have you ever listened to music for more than 3 h per week at a volume which you would need to shout to be heard or, if wearing headphones, someone else would need to shout for you to hear them?”. The response choices for both questions included, “Yes, for more than 5 years”, “Yes, for around 1–5 years”, “Yes, for less than a year?”, “No”, “Do not know”, and “Prefer not to answer”.

### Genome-wide association study (GWAS)

The genotyping was performed using two arrays, the Affymetric UK BiLEVE Axiom and Affymetric UK Biobank Axiom array. About 50,000 samples were genotyped on the Affymetric UK BiLEVE Axiom platform and 450,000 on the UK Biobank Axiom array. The genotypes were augmented by imputation using the Haplotype Reference Consortium^29^. Outliers with high heterozygosity or missingness were excluded^29^. Individuals of self-reported “White British” ancestry and similar genetic ancestry based on principal components were retained for GWAS analysis^29^. GWAS analysis was performed using REGENIE using age, sex, age^2, age × sex, the first 10 principal components, genotype batch, and testing site as covariates^29,30^. An additional study was done using two additional environmental covariates: loud music exposure frequency (#4836) and noisy workplace (#4725), both encoded as ordinal variables.

We identified 168,259 participants responding to the tinnitus question (#4803) after removing those with “do not know” and “prefer not to answer”. We included participants reporting British and Irish white ethnicity, which resulted in the exclusion of 21,116 participants. We excluded 2852 participants reporting “do not know” and “prefer not to answer” for the noisy workplace (#4825) and loud music exposure (#4836) questions. Related individuals were filtered out by excluding one individual in each pair of individuals with a kinship coefficient of greater than 0.0844 (greater than third-degree relationships). The remaining sample (N = 132,438) with non-missing phenotype and quality-controlled genotype data was used for further analysis.

The following filters were applied on genotype and imputed data: a minor allele frequency of > 0.5%, a genotyping rate of 99%, a minor allele count of > 5, a Hardy–Weinberg equilibrium test *p* < 10^–15^, not present in low-present in the low complexity regions, and not involved in the inter-chromosomal LD. For step 1 in the REGENIE, UKB inter-chromosome LD and low-complexity regions were filtered out using the ‘—exclude’ flag. We applied LD pruning (R^2^ = 0.9, window size = 1000, step size = 100) on directly genotyped SNPs, and a total of 471,734 SNP markers were used for step 1. Sex, genotype batch, and testing site were listed as categorical covariates using the “—catCovarList” flag. The size of genotype blocks was set to 1000 for step 1 and 400 for step 2. In step 2, an approximation for the firth correction was used for *p*-values less than 0.01 using the “—firth” and “—approx” flags. Step 2 was performed on 8,357,671 imputed genetic variants achieving quality control. The *p*-value threshold of 5 × 10^–8^ was used to identify genetic associations with tinnitus phenotypes.

Pathway enrichment analysis was performed with FUMA^31^ with the following settings: Maximum *p*-value cutoff of lead SNPs of 1e-5; Maximum p-value cutoff of 1e−4; r^2^ threshold to define independent significant SNPs of 0.8; second R^2^ threshold to define independent significant SNPs of 0.1; UKB release2b 10 k White British for the reference panel population; maximum distance between LD blocks to merge into a locus of 250 kb; distance to genes or functional consequences of SNPs on genes to map of 100 kb. The gene-based analysis was performed using MAGMA (within FUMA). For the gene-based MAGMA analysis, a gene window of 50 kb upstream and 40 kb downstream was used. Otherwise, all settings were left as default. The multiple test correction was performed using the Benjamini–Hochberg procedure per data source tested gene sets, and adjusted *p* < 0.05 was considered a statistically significant association.

## Results

Table 1 presents the demographic details of the sample. In a sample of 132,438 participants with the complete phenotype and genotype data, 38,525 individuals (29.1%) reported any form of tinnitus. Among individuals with tinnitus, 8720 (6.6%) reported they experience tinnitus “most or all of the time”, 3347 (2.5%) reported tinnitus “now a lot of the time”, 11,816 (8.9%) reported tinnitus for “now some of the time”, and 14,642 (11%) reported, “not now, but had in the past”. 93,913 individuals (70.9%) reported no tinnitus experience lasting five minutes or more at a time. Table 1 presents the prevalence of tinnitus with sex, ethnicity, music exposure, noise exposure, and testing sites. The study sample included 61,646 (46.5%) males and 70,792 (53.5%) females. The prevalence of any form of tinnitus was higher in males than females (OR = 0.75, 95%CI = 0.73–0.76, *p* < 10^–123^). The prevalence of “most or all of the time” tinnitus perception was also higher in males than females (OR = 0.51, 95%CI 0.49–0.53, *p* < 10^–10^). Individuals with music exposure (OR = 1.88, 95%CI 1.82–1.92, *p* < 10^–10^) and work-related noise exposure (OR = 2.14, 95%CI 2.08–2.20, *p* < 10^–10^) showed a significantly higher prevalence of any form of tinnitus than their counterparts.

**Table 1:** Prevalence of tinnitus across the experimental variables (N = 132,438).

Experimental variables	Tinnitus				
	No, never	Yes, but not now, but have in the past	Yes, now some of the time	Yes, now a lot of the time	Yes, now most or all of the time
Sex (male > female)**					
Male	41,761 (31.5%)	6878 (5.2%)	5857 (4.4%)	1839 (1.4%)	5311 (4%)
Female	52,152 (39.4%)	7764 (5.9%)	5959 (4.5%)	1508 (1.1%)	3409 (2.6%)
**Ethnicity**					
British White	90,696 (68.5%)	14,134 (10.7%)	11,460 (8.7%)	3258 (2.5%)	8472 (6.4%)
Irish White	3217 (2.4%)	508 (0.4%)	356 (0.3%)	89 (0.1%)	248 (0.2%)
Loud music exposure (none > yes)*					
None	83,616 (63.9%)	11,684 (8.9%)	9697 (7.4%)	2720 (2.1%)	7198 (5.5%)
Yes, less than 1 year	2388 (1.8%)	753 (0.6%)	478 (0.4%)	125 (0.1%)	276 (0.2%)
Yes, 1–5 years	3787 (2.9%)	980 (0.7%)	674 (0.5%)	222 (0.2%)	507 (0.4%)
Yes, more than 5 years	3154 (2.4%)	959 (0.7%)	757 (0.6%)	225 (0.2%)	597 (0.5%)
Work-related noise exposure (none > yes)*					
None	75,372 (57.4%)	9859 (7.5%)	7856 (6%)	2176 (1.7%)	5365 (4.1%)
Yes, less than 1 year	4683 (3.6%)	1139 (0.9%)	719 (0.5%)	206 (0.2%)	464 (0.4%)
Yes, 1–5 years	4430 (3.4%)	1180 (0.9%)	941 (0.7%)	273 (0.2%)	671 (0.5%)
Yes, more than 5 years	8792 (6.7%)	2316 (1.8%)	2168 (1.6%)	665 (0.5%)	2133 (1.6%)
Age (40 > 70 years)**					
40–49	20,228 (15.3%)	3589 (2.7%)	1876 (1.4%)	389 (0.3%)	389 (0.3%)
50–59	30,209 (22.8%)	4675 (3.5%)	3633 (2.7%)	1034 (0.8%)	2439 (1.8%)
60–69	43,043 (32.5%)	6301 (4.8%)	6227 (4.7%)	1903 (1.4%)	5255 (4%)
> 70	433 (0.3%)	77 (0.1%)	80 (0.1%)	21 (0%)	77 (0.1%)

**Chi square *p* < 10^–100^, *Chi square *p* < 10^–10^.

Table 2 presents the prevalence of tinnitus-related distress across the study sample. The study sample included 1211 individuals (0.9% of the overall sample) reporting they were severely annoyed, worried, or upset due to their tinnitus perception. While 6278 (4.8%) reported they were moderate, 18,400 (14%) were slight, and 12,362 (9.3%) were not at all annoyed, worried, or upset due to their tinnitus perception. We compared the prevalence of individuals reporting significant tinnitus-related distress (those who reported “slightly”, “moderately”, and “severely”) and those without tinnitus-related distress. Females reported a significantly higher prevalence of tinnitus-related distress than males (OR = 1.38, 95%CI 1.32–1.44, *p* < 10^–10^). Noise exposure (OR = 0.98, 95%CI 0.94–1.03, *p* = 0.59) did not show a significant association with tinnitus-related distress (OR = 0.98, 95%CI 0.94–1.03, *p* = 0.59), and music exposure revealed a weak association (OR = 0.92, 95%CI 0.87–0.98, *p* = 0.009). Therefore, we did not include them as covariates for the GWAS investigating tinnitus-related distress.

**Table 2:** Prevalence of reaction to tinnitus across the experimental variables.

Experimental variables	Reaction to tinnitus				
	No tinnitus	Not at all	Slightly	Moderately	Severely
Sex (females > males)*					
Male	41,761 (31.6%)	7059 (5.3%)	9188 (7%)	2992 (2.3%)	542 (0.4%)
Female	52,152 (39.5%)	5303 (4%)	9212 (7%)	3286 (2.5%)	669 (0.5%)
**Ethnicity**					
British White	90,696 (68.6%)	12,022 (9.1%)	17,842 (13.5%)	6035 (4.6%)	1169 (0.9%)
Irish White	3217 (2.4%)	340 (0.3%)	558 (0.4%)	243 (0.2%)	42 (0%)
**Loud music exposure**					
None	83,616 (64.1%)	9970 (7.6%)	15,092 (11.6%)	5047 (3.9%)	975 (0.7%)
Yes, less than 1 year	2388 (1.8%)	561 (0.4%)	747 (0.6%)	268 (0.2%)	45 (0%)
Yes, 1–5 years	3787 (2.9%)	752 (0.6%)	1138 (0.9%)	412 (0.3%)	65 (0%)
Yes, more than 5 years	3154 (2.4%)	883 (0.7%)	1103 (0.8%)	431 (0.3%)	103 (0.1%)
**Work-related noise exposure**					
None	75,372 (57.5%)	8095 (6.2%)	12,367 (9.4%)	3912 (3%)	710 (0.5%)
Yes, less than 1 year	4683 (3.6%)	901 (0.7%)	1200 (0.9%)	350 (0.3%)	61 (0%)
Yes, 1–5 years	4430 (3.4%)	978 (0.7%)	1411 (1.1%)	533 (0.4%)	118 (0.1%)
Yes, more than 5 years	8792 (6.7%)	2283 (1.7%)	3238 (2.5%)	1411 (1.1%)	305 (0.2%)
Age (years) (40 > 70 year)**					
40–49	20,228 (15.3%)	2672 (2%)	2929 (2.2%)	951 (0.7%)	183 (0.1%)
50–59	30,209 (22.9%)	3604 (2.7%)	5778 (4.4%)	1876 (1.4%)	434 (0.3%)
60–69	43,043 (32.6%)	6001 (4.5%)	9577 (7.2%)	3409 (2.6%)	584 (0.4%)
> 70	433 (0.3%)	85 (0.1%)	116 (0.1%)	42 (0%)	10 (0.1%)

**Chi square *p* < 10^–88^, *Chi square *p* < 10^–10^.

### GWAS results for tinnitus

**Figure 1:**
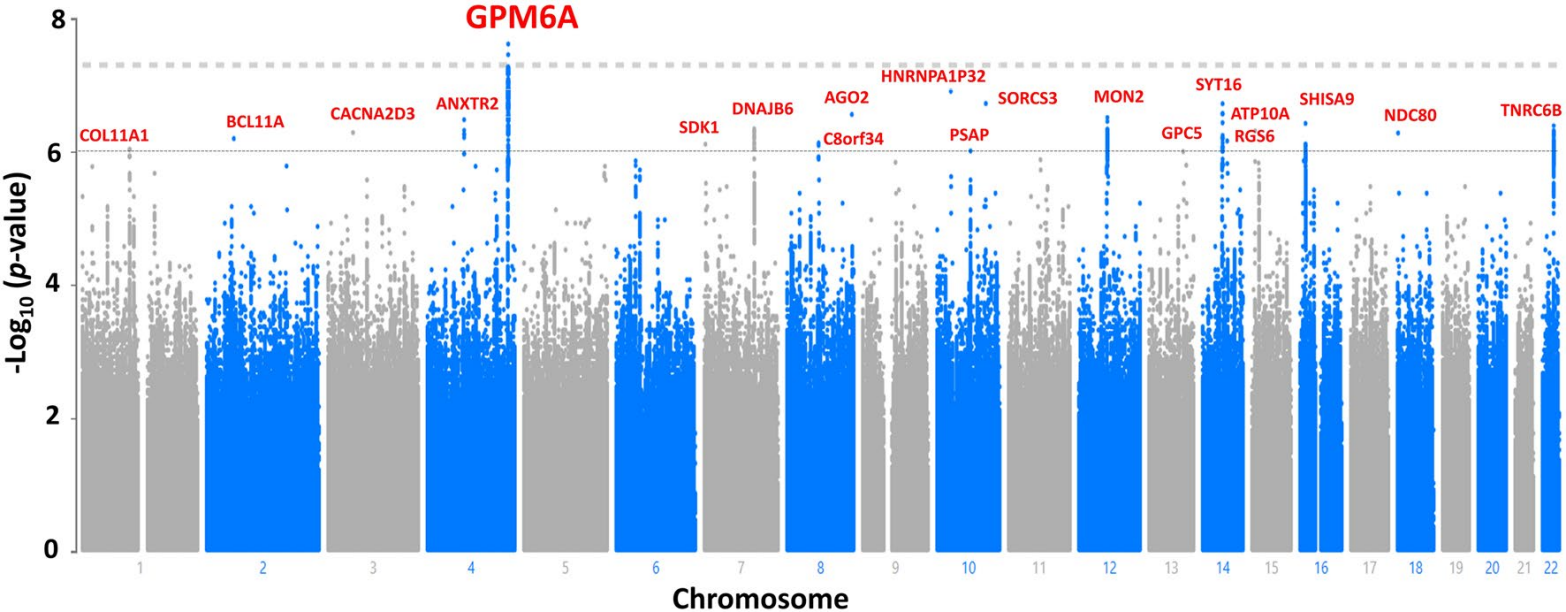
Manhattan plot for the genome-wide association study on tinnitus. The dashed gray line presents the genome-wide significance threshold (*p* < 5E−8), and the dashed black line indicates the suggestive significance threshold (*p* < E−6).

**Figure 2:**
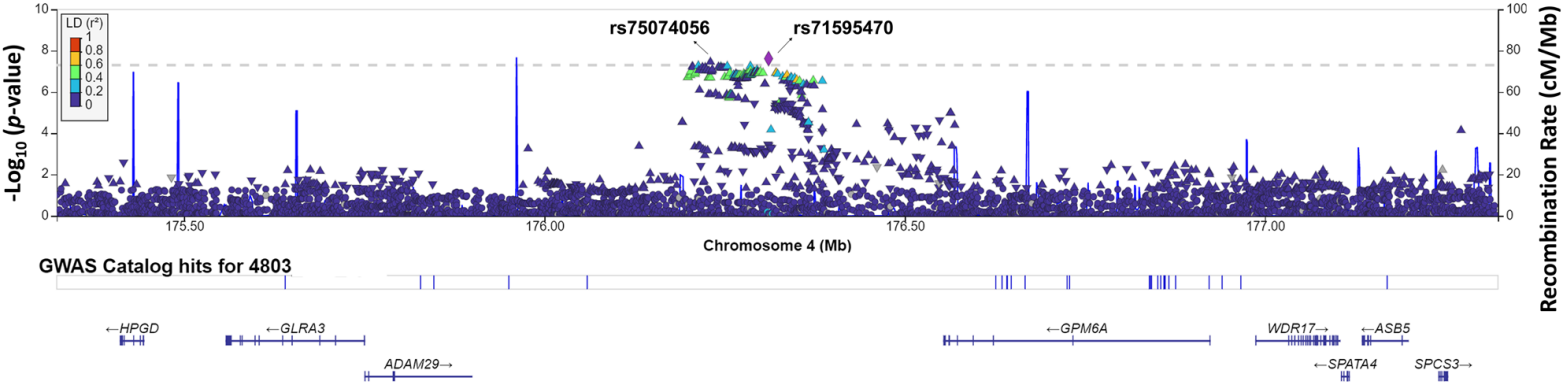
LocusZoom plot of the genomic region in the proximity to *GPM6A* associated with tinnitus. The graph shows the linkage disequilibrium in the region of rs71595470. Each dot is showing the *p*-value for the SNPs in this region. The color bar indicates the level of the linkage disequilibrium.

Figure 1 presents a Manhattan plot for tinnitus, and Table 3 shows the lead SNPs achieving genome-wide suggestive significance of *p*-value < 10^–6^. The GWAS identified one locus, with a lead SNP rs71595470, in the proximity to *GPM6A,* reaching the genome-wide significance with the *p*-value of 2.48E-8 (Fig. 2, Table 3, Supplementary File S1) (Genomic Control λ = 1.12). Another SNP, rs75074056, in proximity to rs71595470, achieved genome-wide significance (Fig. 2). Nineteen independent loci reached suggestive significance (Table 3). The major genes within or near the associated loci are listed in Table 3. Gene-based testing of SNPs summary statistic data identified *PSAP* and *TNRC6* were significantly associated with tinnitus (Supplementary File S1).

**Table 3:** SNPs achieving suggestive significance (*p* < 10^–6^) for tinnitus.

rsID	Chr:pos	Gene	A1/A2	Reference allele	β	A2 freq	p-value
**rs71595470**	**4: 176,310,931**	GPM6A*, ADAM29*	**AATAG/A**	**AATAG**	**0.067**	**0.035**	**2.42E−8**
rs138348879	10: 30,800,837	*HNRNPA1P32*^+^/MAP3K8*	T/C	T	0.085	0.02	1.24E−7
rs72815660	10: 106,614,698	SORCS3**	A/G	A	0.06	0.039	1.89E−7
rs115280365	14: 62,444,770	SYT16**	C/T	C	− 0.06	0.039	1.90E−7
rs2977458	8: 141,576,720	AGO2**	G/T	G	− 0.25	0.301	2.76E−7
rs11174486	12: 62,848,152	MON2*	G/A	G	0.024	0.36	3.08E−7
rs6830642	4: 80,812,744	ANTXR2*	T/C	T	0.033	0.86	3.31E−7
	16: 13,029,711	SHISA9**	G/GA	G	− 0.026	0.705	3.77E−7
	22: 40,560,229	TNRC6B**	GA/G	GA	0.023	0.605	4.06E−7
rs10225195	7: 109,167,243	DNAJB9*	T/C	T	0.022	0.476	4.54E−7
rs112291465	15: 25,874,698	ATP10A*	T/A	T	0.033	0.139	4.88E−7
rs9841474	3: 55,206,208	CACNAD3*	G/A	G	0.027	0.217	5.18E−7
rs145362852	18: 2,600,477	NDC80*	G/A	G	0.069	0.028	5.25E−7
rs145529681	2: 60,073,205	BCL11A*	C/G	C	0.067	0.029	6.37E−7
rs201893107	14: 72,429,274	RGS6**	CTG/C	CTG	0.117	0.01	6.92E−7
	8: 69,546,107	C8orf34**	GT/G	GT	− 0.023	0.647	7.42E−7
rs12155428	7: 3,425,301	SDK1**	C/T	C	0.061	0.033	7.72E−7
rs11164650	1: 103,448,242	COL11A1**	T/G	T	− 0.025	0.248	9.20E−7
rs727415	10: 73,582,752	PSAP**	T/A	T	0.024	0.293	9.77E−7
rs79604213	13: 93,040,866	GPC5**	A/G	A	0.116	0.009	9.98E−7

Significant values are in bold.

**Within a gene body.

*Nearest gene.

^+^Pseudogene.

### FUMA enrichment analysis for tinnitus

We obtained significant results for positional gene sets, transcription factor targets gene sets, and GWAS catalog reported genes (Supplementary File S2). Twenty-three GWAS catalog gene sets revealed a significant association with tinnitus. The significant GWAS catalog gene sets associated with tinnitus included psychiatric and psychological traits, such as bipolar disorder, feeling worried, cognitive ability, fast beta electroencephalogram, and sensation seeking. Metabolic traits, such as wait-to-hip ratio adjusted for BMI, showed significant association with tinnitus. The gene set related to cardiovascular traits, such as hypertension, ischemic stroke, mean arterial pressure, stroke, systolic and diastolic blood pressure (main effect and interaction with smoking and alcohol), and plateletcrit showed significant association with tinnitus. Gene sets related to diuretics, metformin, fenofibrate, and *TNF* inhibitor used for rheumatoid arthritis showed association with tinnitus. The enrichment analysis for differentially expressed genes revealed that the GWAS signals were enriched in genes upregulated in the brain hippocampus (differentially expressed genes-two-sided adjusted *p*-value = 0.006, differentially expressed genes-upregulated adjusted *p*-value = 0.046) and cortex (differentially expressed genes-upregulated adjusted *p*-value = 0.015). (Supplementary Files S1–S2).

### GWAS results for tinnitus-related distress

**Figure 3:**
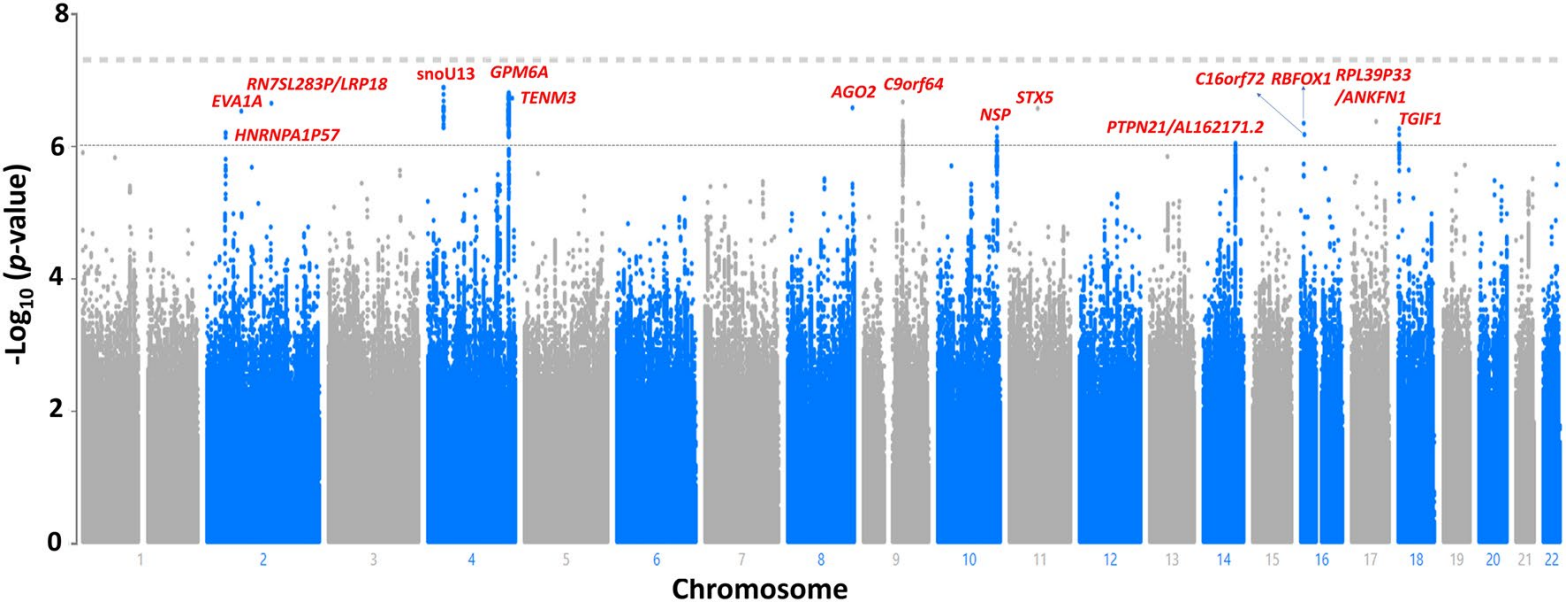
Manhattan plot for the genome-wide association study on tinnitus-related distress. The dashed gray line presents the genome-wide significance threshold (*p* < 5E−8), and the dashed black line indicates the suggestive significance threshold (*p* < E−6).

**Figure 4:**
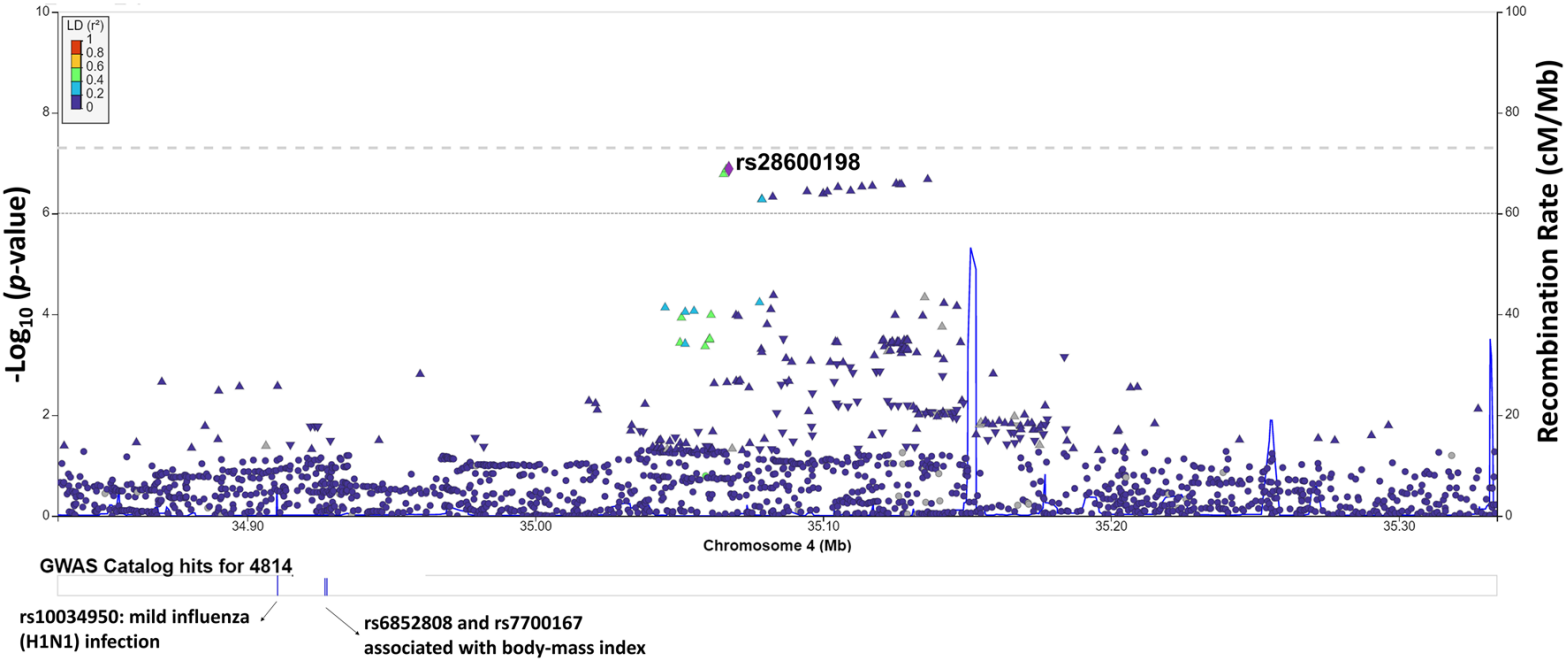
LocusZoom plot of the genomic region associated with tinnitus. The graph shows the linkage disequilibrium in the region of rs28600198. Each dot is showing the *p*-value for the SNPs in this region. The color bar indicates the level of the linkage disequilibrium.

Figure 3 presents a Manhattan plot for tinnitus-related distress, and Table 4 shows the SNPs achieving the suggestive significance. Fifteen independent loci showed suggestive association with tinnitus-related distress (Genomic Control λ = 1.10). However, none of the tested SNPs achieved genome-wide significance. A SNP (rs28600198) in a non-coding RNA gene (*snoU13* on Chromosome 4), achieved the lowest *p*-value (Fig. 4). The gene-based test showed a significant association between *PSAP* and tinnitus-related distress (Supplementary File S1).

**Table 4:** SNPs achieving the suggestive significance (*p* < 10^–6^) for tinnitus-related distress.

rsID	Chr:pos	Gene	A1/A2	Reference allele	β	A2 freq	*p*-value
rs28600198	4: 35,067,243	*snoU13*/ARAP2*	C/T	C	0.064	0.024	1.30E−7
rs113604070	4: 176,291,501	GPM6A*, ADAM29*	T/C	C	0.054	0.034	1.58E−7
rs566169037	4: 183,705,777	TENM3**	T/C	T	0.096	0.011	1.90E−7
	9: 86,553,664	C9orf64*	AT/A	AT	0.022	0.260	2.17E−7
rs572608059	2: 140,770,563	*RN7SL283P*/LRP1B*	…T/G	T	0.142	0.005	2.27E−7
rs2977458	8: 141,576,720	AGO2**	G/T	G	− 0.021	0.301	2.66E−7
	11: 62,596,218	STX5**	T/TAAAC	T	− 0.022	0.565	2.71E−7
rs139919044	2: 75,699,744	EVA1A**	C/T	C	0.095	0.011	2.99E−7
rs77782297	17: 54,624,719	*RPL39P33*/ANKFN1*	A/C	A	0.069	0.020	4.27E−7
rs554121008	16: 7,646,138	RBFOX1**	C/A	C	− 0.030	0.886	4.53E−7
rs6482934	10: 129,362,692	NPS*	T/C	T	− 0.050	0.963	5.31E−7
rs9949773	18: 3,436,449	TGIF1**	G/A	G	0.020	0.656	5.49E−7
	2: 41,551,500	*HNRNPA1P57*^+^/SLC8A1*	T/TA	T	0.054	0.032	6.36E−7
rs146976502	16: 9,174,460	C16orf72*	C/T	C	0.116	0.007	6.71E−7
rs2778938	14: 88,972,303	*PTPN21*/AL162171.2**	A/G	A	0.019	0.656	9.13E−7

**Within a gene body.

*Nearest gene.

^+^Pseudogene.

### FUMA enrichment analysis for tinnitus-related distress

The GWAS catalog gene sets related to anxiety and stress-related disorders showed four (out of 15) overlapping genes with the tinnitus gene set. Heel bone mineral density, cutaneous systemic scleroderma, molar-incisor hypomineralization, and lumbar disc degeneration showed significantly overlapped genes with tinnitus. The GWAS signals were enriched in genes differentially expressed in the brain and spinal cord (cervical c-1) (differentially expressed genes-upregulated adjusted *p*-value = 0.039). (Supplementary Files S1 and S3).

## Discussion

The present study conducted a genome-wide association analysis to identify SNPs associated with tinnitus and tinnitus-related distress using the UK Biobank database. A SNP close to *GPM6A* achieved genome-wide significance, and 19 independent genetic loci showed suggestive association with tinnitus. Tinnitus-related distress showed suggestive association with 15 independent genomic loci. The gene-based test identified *PSAP* was associated with tinnitus and tinnitus-related distress, and *TNRC6* was associated with tinnitus. The enrichment analysis identified psychiatric, cardiovascular, metabolic, hematological, and pharmacogenomic genomic signatures associated with tinnitus and tinnitus-related distress. The differential gene expression enrichment analysis revealed that the GWAS signals for tinnitus were enriched in genes upregulated in the hippocampus and cortex. They were enriched in genes differentially expressed in the brain and spinal cord for tinnitus-related distress (cervical c-1). Table 5 presents the summary statistics for SNPs associated with tinnitus by Wells et al. and Clifford et al.^23,24^. The differences in the results could be attributed to the sample selection criteria and GWAS methods.

**Table 5:** The summary statistics for the top SNPs associated with tinnitus by Wells et al.^24^ and Clifford et al.^23^.

SNP rsID	Chr:pos	A1	A2	Current study			Clifford et al.^23^			Wells et al.^24^		
				β	A1 Freq	*p-value*	β	A1 Freq	*p-value*	β	A1 Freq	*p-value*
**rs143424888**	**1:103,456,996**	**C**	**CACGTGATCT**	**− 0.021**	**0.508**	**2.8E−6**	**− 0.023**	**0.510**	**8.5E−9**	**− 0.006**	**0.508**	**9.0E−6**
rs17249745	4:102,547,366	A	G	− 0.049	0.967	2.3E**−**4	− 0.072	0.967	1.3E**−**9	− 0.018	0.968	1.0E**−**5
**rs553448379**	**6:43,288,656**	**T**	**TA**	− **0.022**	**0.385**	**1.6E−6**	− **0.023**	**0.386**	**2.1E−8**	− **0.007**	**0.384**	**4.6E**−**7**
**rs11249981**	**8:10,147,398**	**C**	**T**	− **0.018**	**0.448**	**6.2E−5**	− **0.022**	**0.447**	**4.6E−8**	− **0.007**	**0.449**	**1.2E**−**6**
rs72815660	10:106,614,698	A	G	− 0.060	0.961	1.8E**−**7	− 0.060	0.960	9.7E**−**9	− 0.014	0.961	4.1E**−**5
rs11174489	12:62,852,271	G	A	− 0.023	0.625	5.4E**−**7	− 0.024	0.625	3.8E**−**9	− 0.006	0.626	1.1E**−**5

Significant values are in bold.

rs143424888, rs553448379, and rs11249981 were replicated in the Million Veteran Program sample by Clifford et al.^23^. SNP rsID presents the rsID of the single nucleotide polymorphism (SNP). Chr:por presents the chromosomal number and position of SNP, A1 is the frequency is of A1 allele, and A2 is the frequency of A2 allele, beta presents the regression correlation coefficient, and p-value presents the observed p-value of the regression coefficient.

### Tissue-specific enrichment of the GWAS signals in the hippocampus and cortex

The interplay between auditory and non-auditory structures following traumatic events such as noise exposure plays an essential role in tinnitus perception^32^. In epidemiological studies, traumatic noise and music exposure are consistently associated with tinnitus^1–3,12^. Noise-induced cochlear deafferentation and hyperactivity in the central auditory pathway were studied as putative mechanisms underlying tinnitus perception^33^. Past studies concluded that hyperactivity in the auditory structure plays a crucial role in tinnitus perception. In contrast, the role of the non-classical auditory structures is limited to regulating tinnitus-related distress^33^. However, recent studies highlighted the critical role of the non-auditory structures, such as the hippocampus, amygdala, and cingulate cortex, in generating and maintaining tinnitus perception^32,34^. The amygdala and hippocampus receive input from the medial geniculate body in the thalamus and can interact with the auditory pathway (e.g.,^35^). The failure to inhibit the hyperactivity generated in the auditory pathway by non-classical auditory structures (such as the limbic system) is a putative mechanism underlying tinnitus perception^36^. Consistent with this hypothesis, recent neuroimaging studies indicate that the hippocampus and parahippocampus in the limbic system are associated with tinnitus (e.g.,^37^). The enrichment of the GWAS signals in the hippocampus and cortex observed in the present study (Supplementary File S1) highlighted their importance in tinnitus perception.

### SNPs associated with tinnitus and tinnitus-related distress

*GPM6A* is the closest gene to the genomic region, with a lead SNP rs71595470 achieving genome-wide significance (Fig. 2). SNPs (rs183819925 and rs76744071) in the vicinity of the region were associated with a cognitive decline rate^38^. Several SNPs in *GPM6A* body revealed association with cognitive ability (rs13136969, rs6553899), schizophrenia (rs7673823, rs13142920, rs62334820, rs2333321, rs1106568; rs6846161), depression (rs6818081), neuroticism (rs72704531, rs17611770), and educational attainment (rs1814701, rs17598675, rs4146675)^39,40^. Tinnitus often accompanies psychiatric comorbidities such as anxiety, depression, cognitive dysfunction, and suicidal thoughts^41,42^. The genomic region containing a lead SNP in the vicinity of *GPM6A* might lie at the crossroad between tinnitus and psychiatric comorbidities.

*GPM6A* is a protein coding gene belonging to the tetraspan proteolipid protein family that encodes neural glycoprotein M6a^43,44^. *GPM6A* plays an essential role in neural growth by functioning as an edge membrane antigen to regulate neurite outgrowth in the cerebellum, cortex, and hippocampus neurons^43,44^. Post-translational modification with phosphorylation of tyrosine 251 at the C-terminus of M6a is essential for neuritogenesis in hippocampal neurons^45^. M6a colonizes at the glutamatergic excitatory presynaptic buttons and with vesicular glutamate transporter in the mossy fiber axon terminals^46^. M6a is localized in the myelin sheath, interacting with > 20 myelin proteins, and is essential for regulating post-synaptic activities (e.g.,^47^). The inefficient regulation of M6a might contribute to glutamate-related neural excitotoxicity, which is associated with tinnitus perception (e.g.,^48^).

Non-synonymous SNPs in *GPM6A* are associated with protein instability, making M6a non-functional in neurons^45,49^. M6a expression positively correlates with synaptic counts in hippocampal neurons^49^. M6a is also important for neural spine formation^49^. The dendritic spine formation is required for normal synaptic development and functioning. The abnormal synaptic functioning is implemented in psychiatric conditions (e.g.,^50^). *GPM6A* is associated with psychiatric traits, such as bipolar diseases, schizophrenia, depression, Alzheimer's disease, and claustrophobia (e.g.,^51–55^). M6a is associated with processing chronic stress in animal models, with chronic stress negatively correlated with gpm6a mRNA levels in the hippocampus^56,57^. These findings are consistent with a human study comparing *GPM6A* mRNA levels in the hippocampus in patients suffering from depression who committed suicide^54^. Together with the GWAS signals upregulated in the hippocampus, our results suggest that inefficient regulation of the genomic region associated with tinnitus involving *GPM6A* in the hippocampal neurons could influence tinnitus perception.

Another genomic region involving *SHISA9* showed suggestive association with tinnitus. SNPs within the region are associated with neuroticism (rs275401, rs12926477), bipolar disorder (rs12935276), depression (rs7200826), and intelligence (rs62028752)^56,57^. *SHISA9* is a protein-coding gene that encodes an auxiliary subunit of the AMPA-type glutamate receptors, which are highly expressed in the hippocampus dentate gyrus, cortex, and olfactory bulb^58^. *SHISA9* can modulate the short-term plasticity of excitatory synapses^59^. *SHISA9* is associated with schizophrenia, left ventricular hypertrophy, and tobacco use disorder^60^. Tinnitus is associated with reduced diastolic and systolic left ventricular mass and volume^61^. Tobacco smoking is consistently associated with tinnitus in large epidemiological studies (e.g.,^1–3^). In summary, the genomic region involving *SHISA9* might influence synaptic plasticity contributing to tinnitus perception.

The genomic loci involving *PSAP* and *TNRC6B* showed suggestive association with tinnitus. The gene-based test identified associations between tinnitus and *PSAP*, and *TNRC6B*. *PSAP* encodes prosaposin, which is required for the catabolism of glycosphingolipids^62^. *PSAP* regulates the cochlear innervation patterns in the organ of the Corti^63^. Mutation in *PSAP* can cause prelingual profound sensorineural hearing loss^64^. *TNRC6B* is a protein-coding gene involved with the RNA interference machinery^65^. Mutations in *TNRC6B* are associated with childhood hearing loss, speech and language delay, fine-motor delay, autism traits, attention deficit, hyperactivity disorders, and musculoskeletal phenotypes^66^. *TNRC6B* can interact with Argonaute (AGO) family proteins to trigger mRNA decay in the cytoplasm^67^. We obtained SNPs in *AGO2* and *TNRC6B* showing association with tinnitus. The interplay between *AGO2* and *TNRC6B* might influence regulatory RNA mechanisms contributing to tinnitus. Further research is needed to evaluate these suggestive associations with tinnitus.

Tinnitus-related distress revealed suggestive associations with 15 SNPs (Table 4). A SNP close to *ARAP2* achieved the lowest *p*-value. *ARAP2* is essential for Akt signaling, glycolysis, and sphingolipid metabolisms^68,69^. *ARAP2* is associated with impaired regulation of emotions, stress, depression, and bipolar disease^70,71^. The genomic region close to *GPM6A* associated with tinnitus revealed a suggestive association with tinnitus-related distress (Table 4). A SNP in *TENM3*, a gene involved with synaptic architecture development^72,73^, achieved suggestive significance. *TENM3* is associated with schizophrenia and autoimmune disorders^74,75^. Our findings highlighted the polygenic architecture underlying tinnitus-related distress.

### Enrichment analysis for the GWAS catalog reported genes

Genes associated with tinnitus were enriched in the gene sets for sensation seeking, psychiatric conditions, cardiovascular diseases, metabolic conditions, and response to pharmacological agents (Supplementary File S1). Sensation seekers might engage in risky auditory behaviors putting them at higher risk for acquiring tinnitus by exposure to intense sound levels and toxic chemicals in recreational and occupational settings (e.g.,^76^). Psychiatric conditions are common comorbidities associated with tinnitus. About 50% of clinical patients suffering from tinnitus report psychiatric comorbidities, such as depression, somatization, anxiety, and bipolar disorder^77^. Fast beta activity in electroencephalogram is associated with psychiatric traits, mental subnormality, major depression, and alcohol use disorder^78^. The fast beta activity in electroencephalogram is used as an endophenotype of mental subnormality to identify genetic variants associated with disinhibitory traits^79–81^. Tinnitus is associated with increased beta activity in the thalamic region^82^. The common genes between tinnitus, psychiatric conditions, and fast beta activity in electroencephalogram might present a comorbid genetic underpinning among these traits.

Cardiovascular diseases are known risk factors for tinnitus (e.g.,^1–3^). Hypertension, stroke, mean arterial pressure, and diastolic and systolic blood pressure gene sets revealed significant enrichment with tinnitus. The gene sets associated with the interaction between diastolic blood pressure and smoking, and interaction between diastolic and systolic blood pressure and alcohol consumption were significantly enriched in tinnitus. Smoking has consistently been associated with tinnitus, while the relationship between alcohol consumption and tinnitus remains elusive^83^. The gene sets related to body mass index revealed an association with tinnitus. Obesity is associated with a higher risk for tinnitus^84^. Besides, a wide range of ototoxic drugs could trigger tinnitus (e.g.,^85^). A recent GWAS obtained a suggestive association between Cisplatin-induced tinnitus and *OTOS* (rs7606353) highlighting a pharmacogenetic component to tinnitus^86^. In the present study, the gene sets associated with diuretics, fenofibrate, tumor necrosis factor-alpha inhibitor, and metformin showed significant enrichment with tinnitus. Exposure to diuretics could disrupt the blood supply to stria vascularis and could induce transient ischemia allowing the toxic chemicals to pass through the cochlear barrier and inflicting irreversible damage to the organ of Corti, which could trigger tinnitus^87^. Fenofibrate is a pharmacological agent used for treating hypertriglyceridemia^88^, which is associated with tinnitus^15^. Tumor necrosis factor-alpha signaling is implemented in noise-induced tinnitus and hearing loss, and its inhibition might have therapeutic value for tinnitus^89^. Metformin is a therapeutic agent for treating type 2 diabetes^90^. The effect of metformin on tinnitus remains elusive. Metformin users might exhibit reduced vestibular schwannoma growth^91^, while some studies reported tinnitus and auditory symptoms as potential side effects of metformin^92,93^. In summary, our results suggest the pharmacogenetic component underlying tinnitus. Further research is required to identify the genetic variants underlying the interaction between tinnitus and pharmaceutical agents.

Gene sets associated with anxiety and stress-related disorders, cutaneous systematic scleroderma, heel bone mineral density, molar-incisor hypomineralization, and lumbar disc degeneration revealed significant enrichment with tinnitus-related distress. The association between tinnitus-related distress and anxiety and stress-related disorders highlighted common genetic underpinning between these comorbid conditions. Scleroderma is associated with a higher risk of tinnitus, hyperacusis, hearing loss, and abnormal speech perception^94,95^. The relationship between bone mineral density and tinnitus remains elusive. Reduced bone mineral density is associated with a higher risk for hearing loss among postmenopausal women^96^, and tinnitus is higher in patients with osteoporosis^97^. The relationships between tinnitus-related distress and molar-incisor hypomineralization and lumbar disc degeneration remain elusive. Further research is needed to identify the epidemiological risk factors underlying tinnitus-related distress.

### Experimental caveats

The present study lacks an independent sample for the replication analysis. We utilized environmental covariates for conducting the GWAS analysis which resulted in losing some sample size and statistical power (Supplement File S1—Fig. 13). Besides, the present study used single questions to define tinnitus phenotypes, which could not efficiently quantify the biological processes underlying tinnitus and tinnitus-related distress. Tinnitus subphenotypes (e.g., noise-induced tinnitus, drug-induced tinnitus, psychometric features of tinnitus) remained unassessed in the present study. Deep phenotyping is required to quantify the multidimensional phenomenological reality of tinnitus and tinnitus-related distress. Environmental covariates used in the GWAS (such as noise and music exposures) were quantified with single questions. A comprehensive assessment of the environmental factors might yield greater precision.

## Summary

We conducted a GWAS on the UK Biobank database (N = 132,438) to obtain SNPs associated with tinnitus and tinnitus-related distress. A genomic region containing SNP (rs71595470) near *GPM6A* revealed a significant association with tinnitus, and 19 SNPs showed suggestive associations with tinnitus. We obtained fifteen SNPs associated with tinnitus-related distress. The enrichment analysis with FUMA identified 23 gene sets associated with tinnitus. These gene sets included psychiatric conditions, cardiovascular diseases, metabolic conditions, and response to pharmaceutical agents. The enrichment analysis revealed association between tinnitus-related distress and anxiety and stress-related disorders, systemic scleroderma, and other conditions. The GWAS signals collectively enriched in hippocampus and cortex for tinnitus, and were enriched in brain, spinal cord, and cervical C-1 for tinnitus-related distress. In summary, our study highlighted a polygenic architecture underlying tinnitus and tinnitus-related distress.

## Supplementary Information

Supplementary Legends.

Supplementary Figures.

Supplementary Tables.

Supplementary Tables.

## Data Availability

The study used the UK Biobank database. The database is publicly available through the UK Biobank website: https://www.ukbiobank.ac.uk/.

## Abbreviations

GWAS: Genome-wide association study
SNP: Single nucleotide polymorphism
