# Supplementary material for: A genome-wide association study of tinnitus reveals shared genetic links to neuropsychiatric disorders
Source: Sci Rep. 2022 Dec 29;12:22511. doi: 10.1038/s41598-022-26413-6 (PMC9800371; doi:10.1038/s41598-022-26413-6)
Supplement: Supplementary file 1 — Supplementary Legends. [file 41598_2022_26413_MOESM1_ESM.docx]

Supplement File S1: The results of the GWAS for tinnitus

- Figure 1: A schematic diagram showing the subject selection criteria and their influence on the sample size.
- Figure 2: LocusZoom results for tinnitus
- Figure 3: Manhattan plot for the gene-based test for tinnitus
- Figure 4: Tissue specificity (GTEX_V8) analysis for tinnitus
- Figure 5: Quantile-Quantile plots of expected and observed *p*-values (converted on a -log10 (*p*-value) scale) for the genome-wide association study model for tinnitus
- Figure 6: Results of the enrichment analysis for tinnitus
- Figure 7: LocusZoom results for tinnitus-related distress
- Figure 8: Manhattan plot for the gene-based test for tinnitus-related distress
- Figure 9: Tissue specificity (GTEX_V8) analysis for tinnitus-related distress
- Figure 10: Results of the enrichment analysis for tinnitus
- Figure 11: Quantile-Quantile plots of expected and observed *p*-values (converted on a -log10 (*p*-value) scale) for the genome-wide association study model for tinnitus-related distress
- Figure 12: A scatter plot between the genomic PCA1 and PCA2 for individuals reporting tinnitus and no tinnitus.
- Figure 13: GWAS power calculation

Supplement File S2: The results of the FUMA analysis for tinnitus

- GS: The table presents the gene sets associated with tinnitus.
- gtex_v8_ts_DEG: The table presents the results of the tissue enrichment analysis for tinnitus. The differentially expressed genes are enriched in the hippocampus and cortex.
- Gene-based test: The table presents the results of the gene-based test.
- SNPs: The table presents the results of the GWAS for tinnitus for SNPs achieving the *p*-values <E-5.
- IndSigSNPs: The table presents the independent significant SNPs associated with tinnitus.

Supplement File S3: The results of the FUMA analysis for tinnitus-related distress

- GS: The table presents the gene sets associated with tinnitus-related distress.
- gtex_v8_ts_DEG: The table presents the results of the tissue enrichment analysis for tinnitus-related distress. The differentially expressed genes are enriched in the hippocampus and cortex.
- Gene-based test: The table presents the results of the gene-based test for tinnitus-related distress.
- SNPs: The table presents the results of the GWAS for tinnitus-related distress for SNPs achieving the *p*-values <E-5.
- IndSigSNPs: The table presents the independent significant SNPs associated with tinnitus-related distress.
