# Supplementary material for: A genome-wide association study of tinnitus reveals shared genetic links to neuropsychiatric disorders
Source: Sci Rep. 2022 Dec 29;12:22511. doi: 10.1038/s41598-022-26413-6 (PMC9800371; doi:10.1038/s41598-022-26413-6)
Supplement: Supplementary file 2 — Supplementary Figures. [file 41598_2022_26413_MOESM2_ESM.docx]

**Figure 1: A schematic diagram showing the subject selection criteria and their influence on the sample size.**


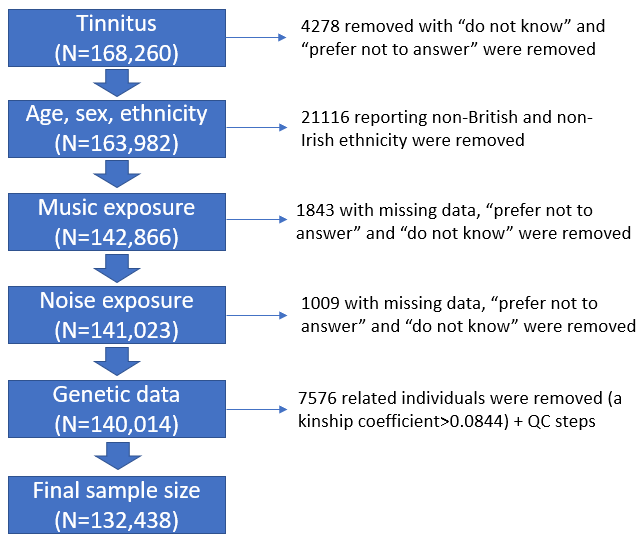


**Figure 2. LocusZoom: TINNITUS (Data-field: 4803)**

Figure 2A: Chr: position: 4: 176,310,931 (GMP6A)
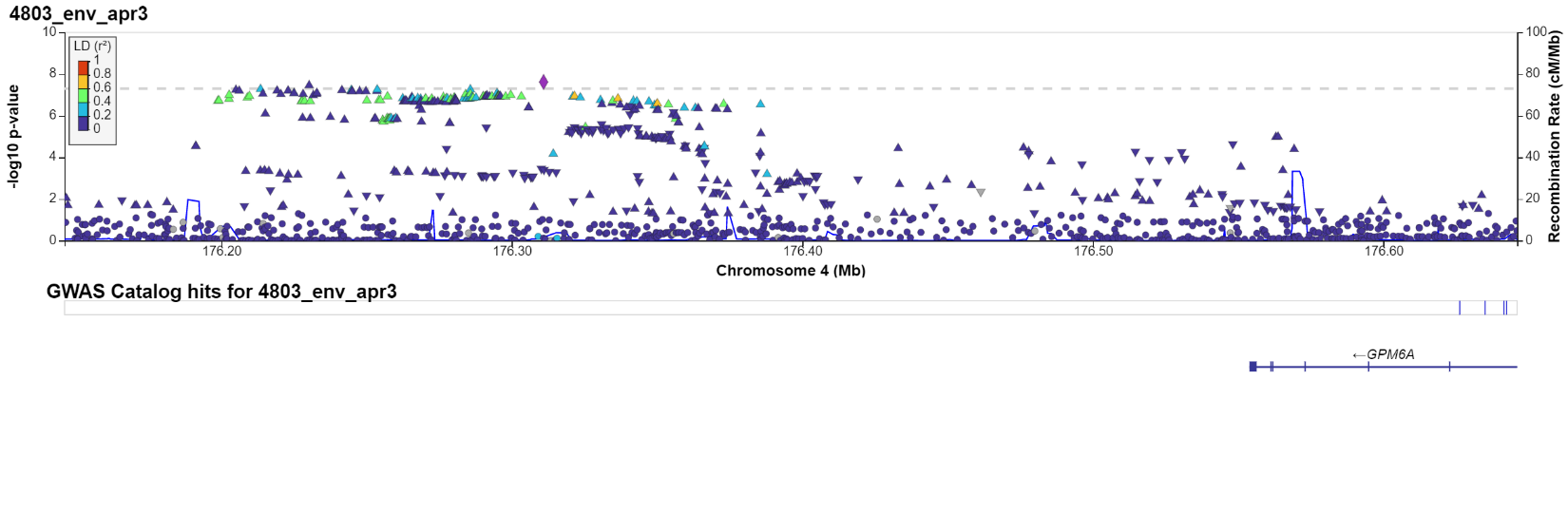


Figure 2B: Chr: position: 10: 30,800,837 (HNRNPA1P32)


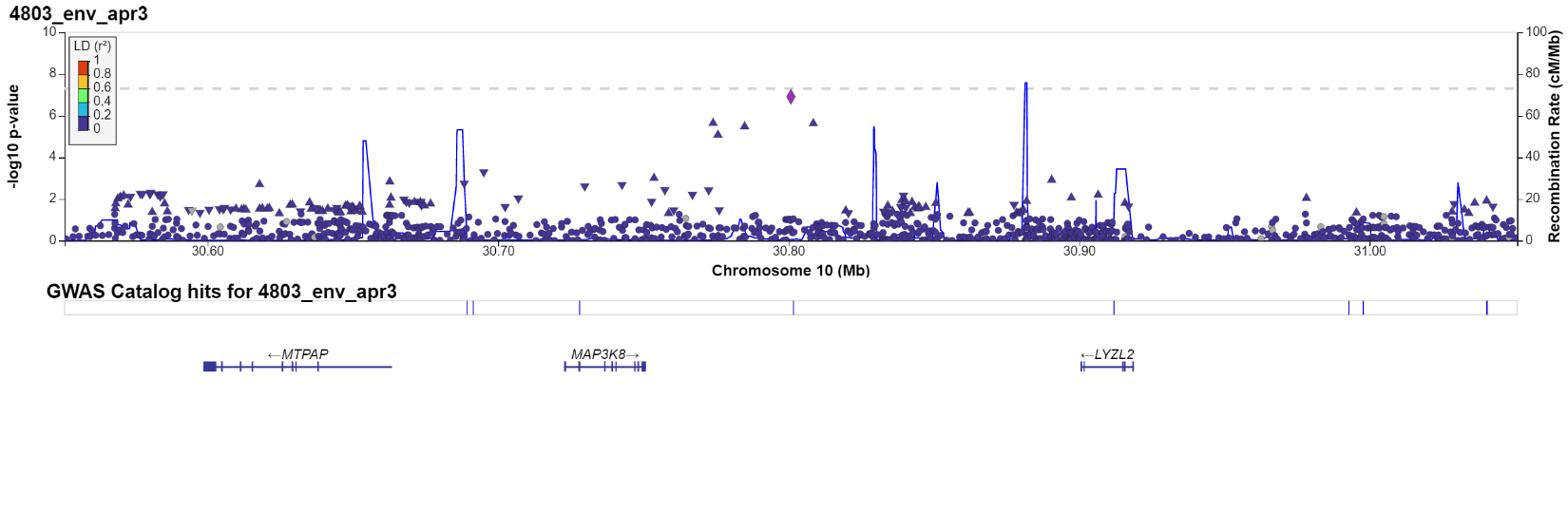


Figure 2C: Chr: position: 10: 106,614,698 (SORCS3)


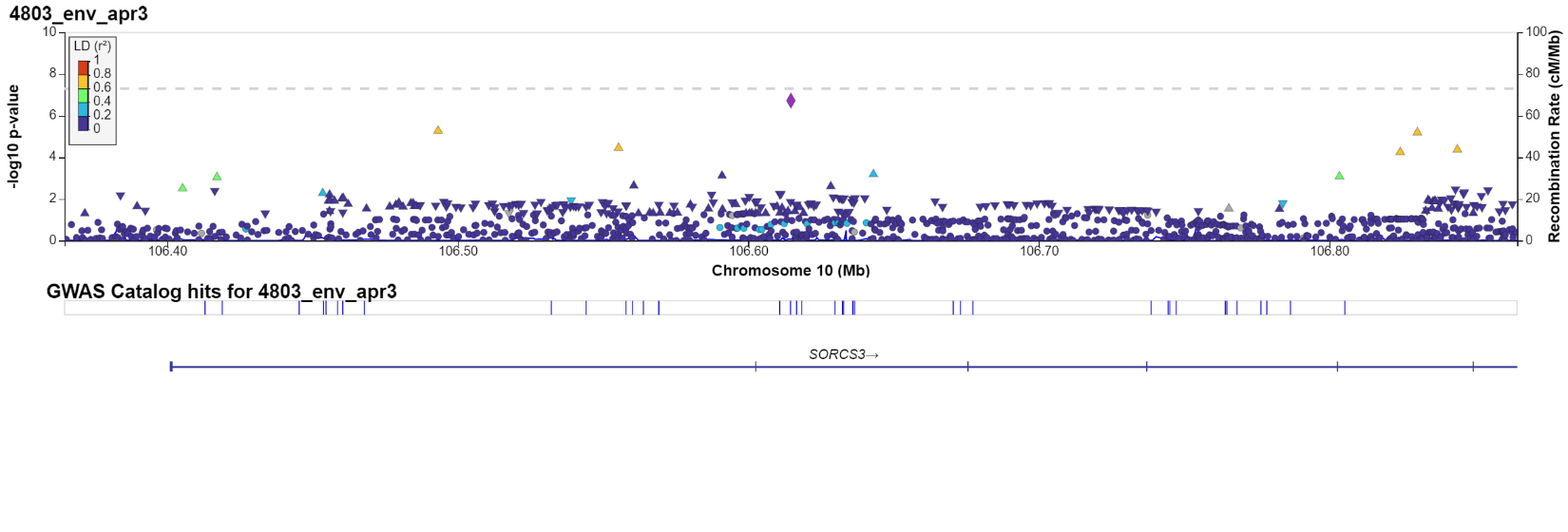


Figure 2D: Chr: position: 14: 62,444,770 (SYT16)


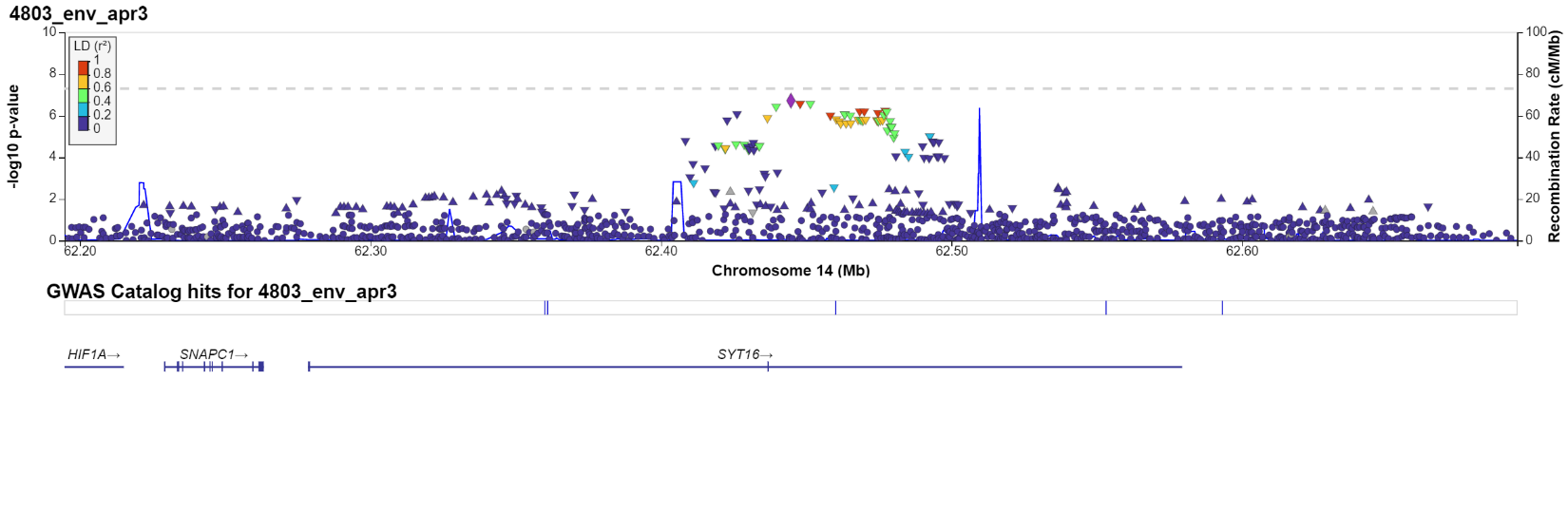


Figure 2E: Chr: position: 8: 141,576,720 (AGO2)


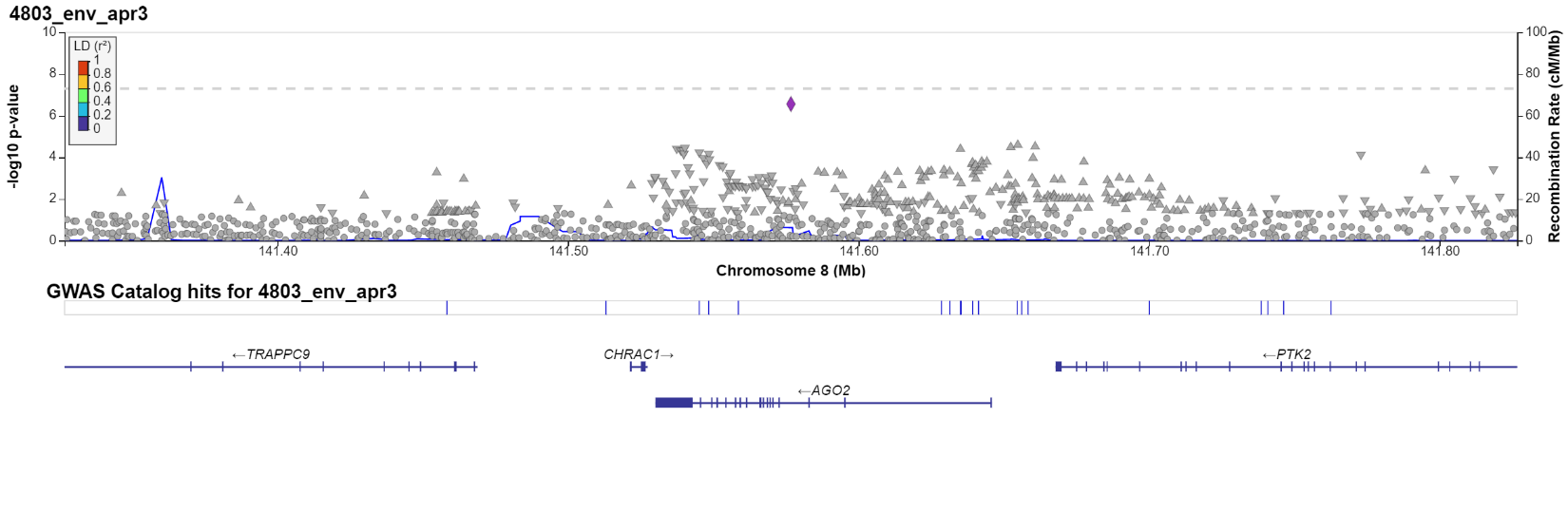


Figure 2F: Chr: position: 12: 62,848,152 (MON2)


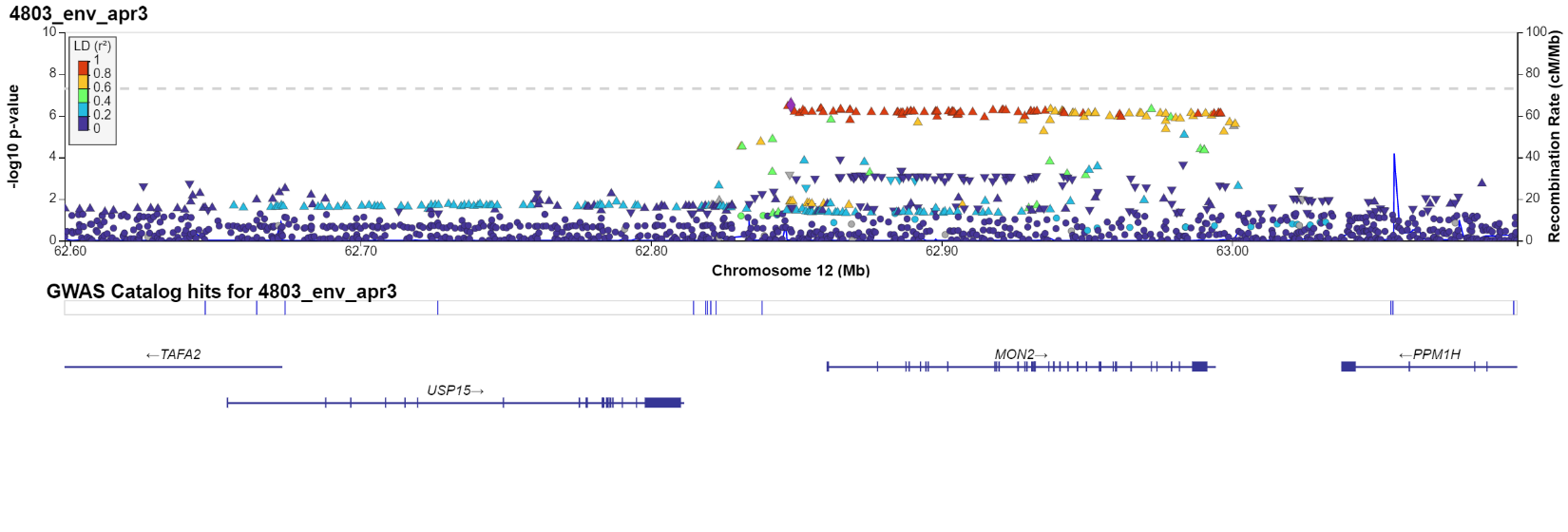


Figure 2G: Chr: position: 4: 80,812,744 (ANTXR2)


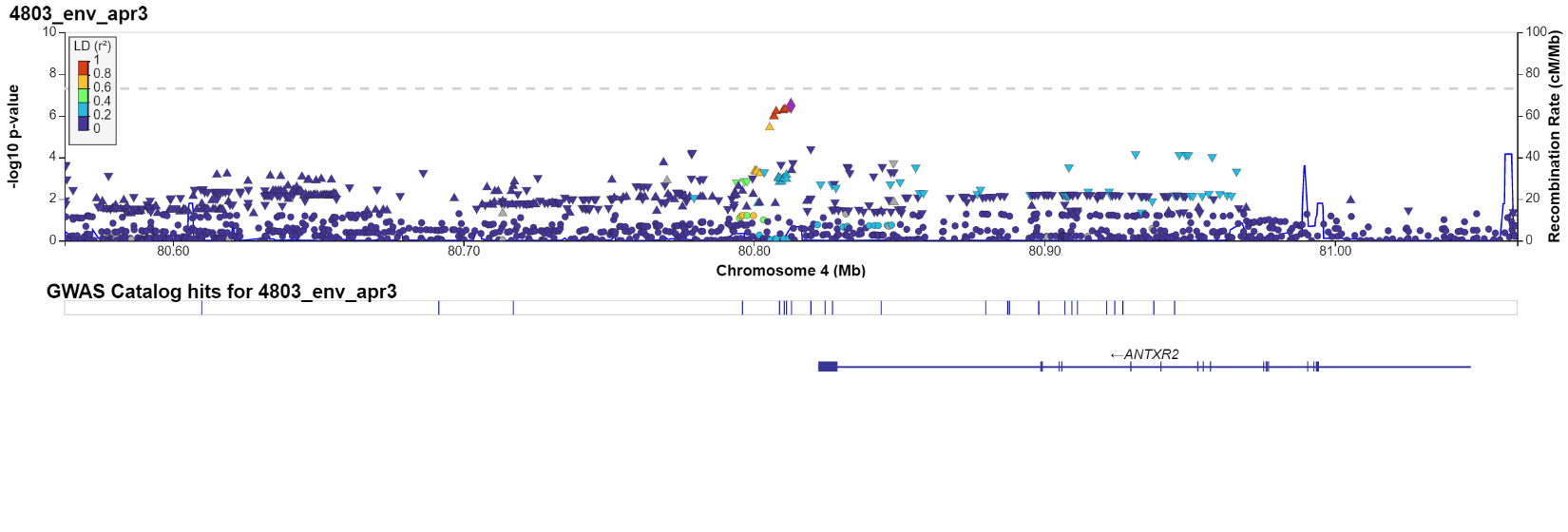


Figure 2H: Chr: position: 16: 13,029,711 (SHISA9)


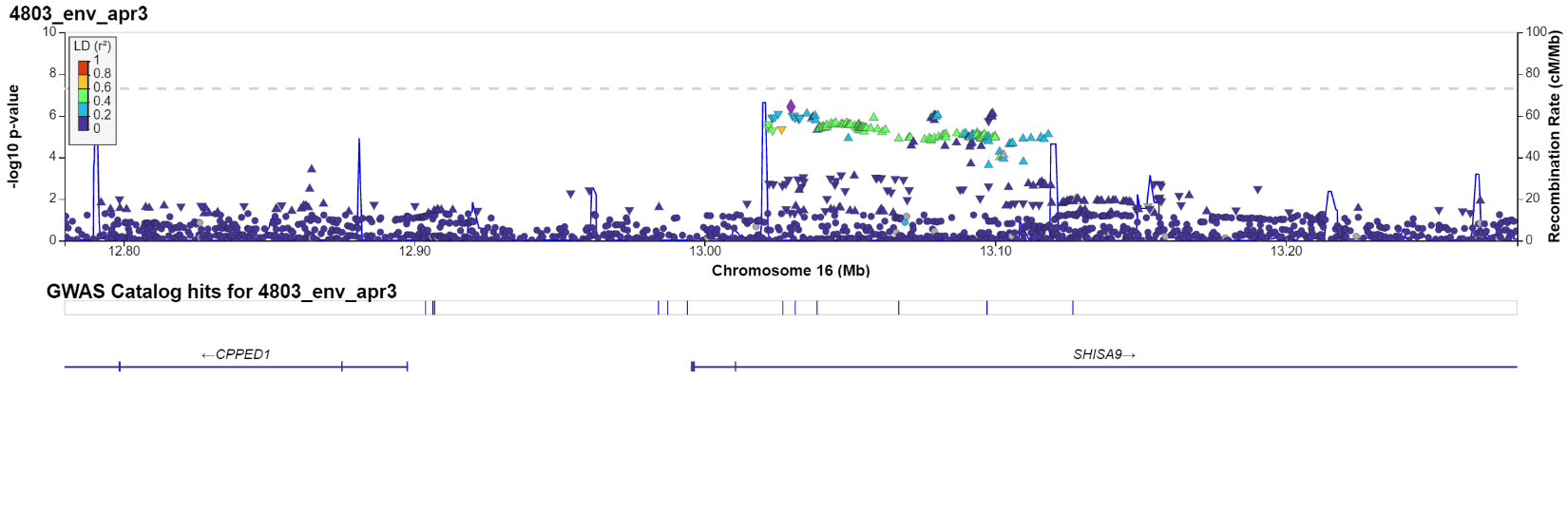


Figure 2I: Chr: position: 22: 40,560,229 (TNRC6B)


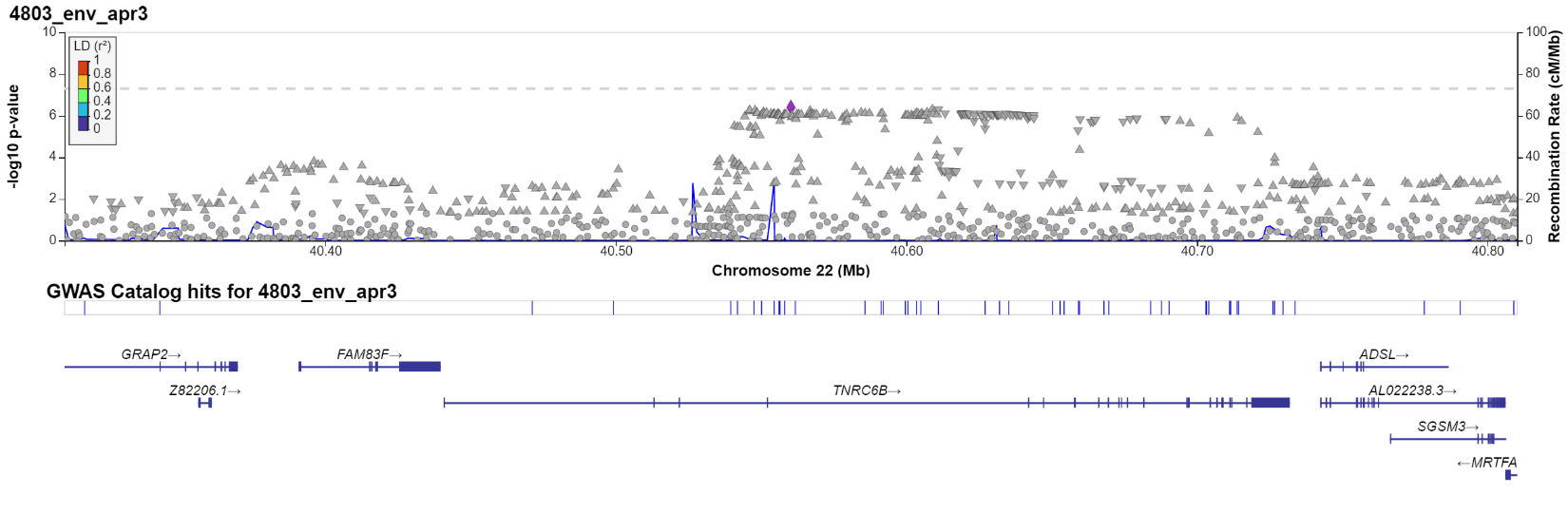


Figure 2J: Chr: position: 7: 109,167,243 (DNAJB9*)


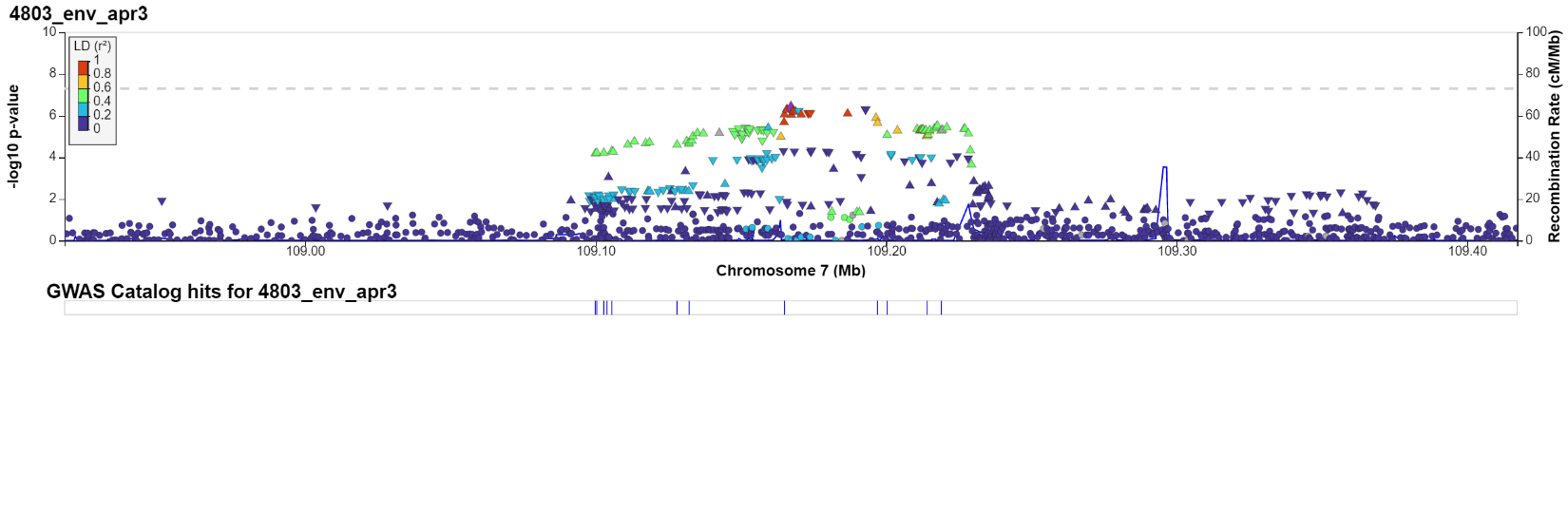


Figure 2K: Chr: position: 15: 25,874,698 (ATP10A)


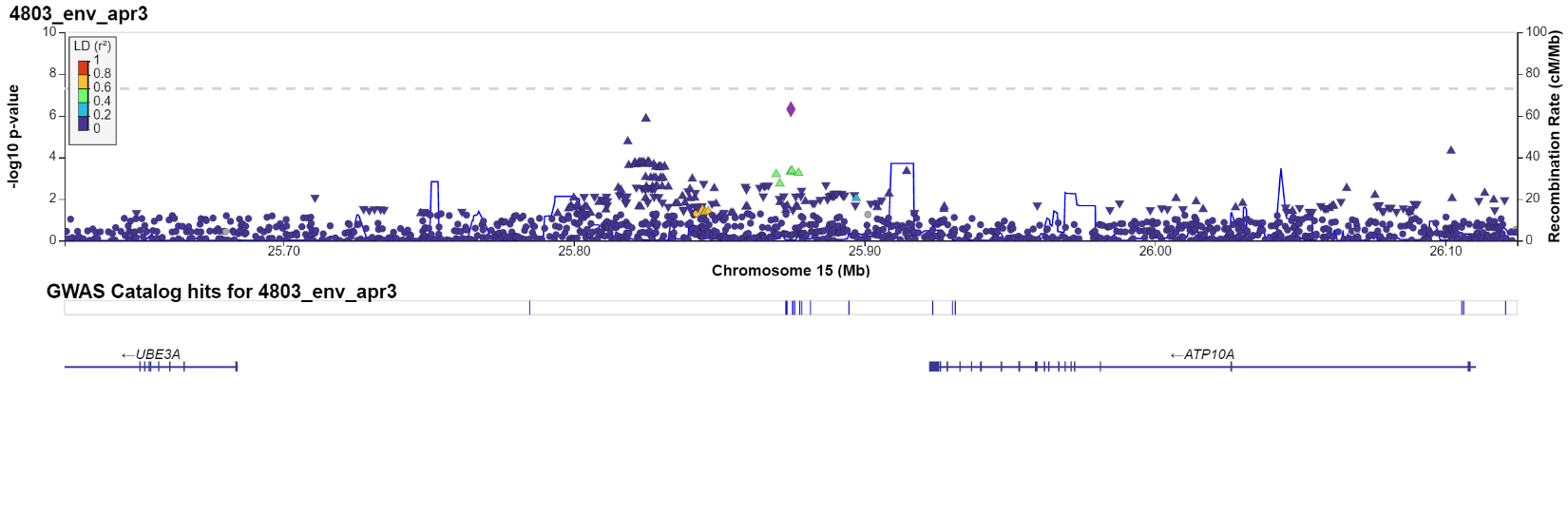


Figure 2L: Chr: position: 3: 55,206,208 (CACNA2D3)


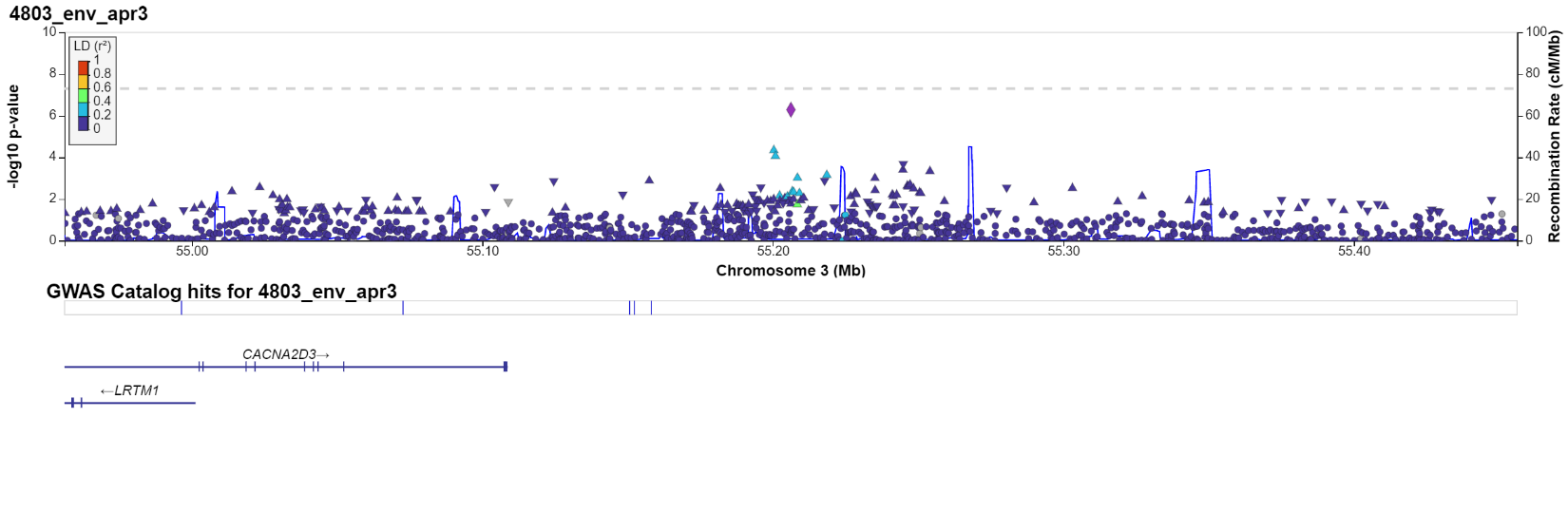


Figure 2M: Chr: position: 18: 2,600,477 (NDC80)


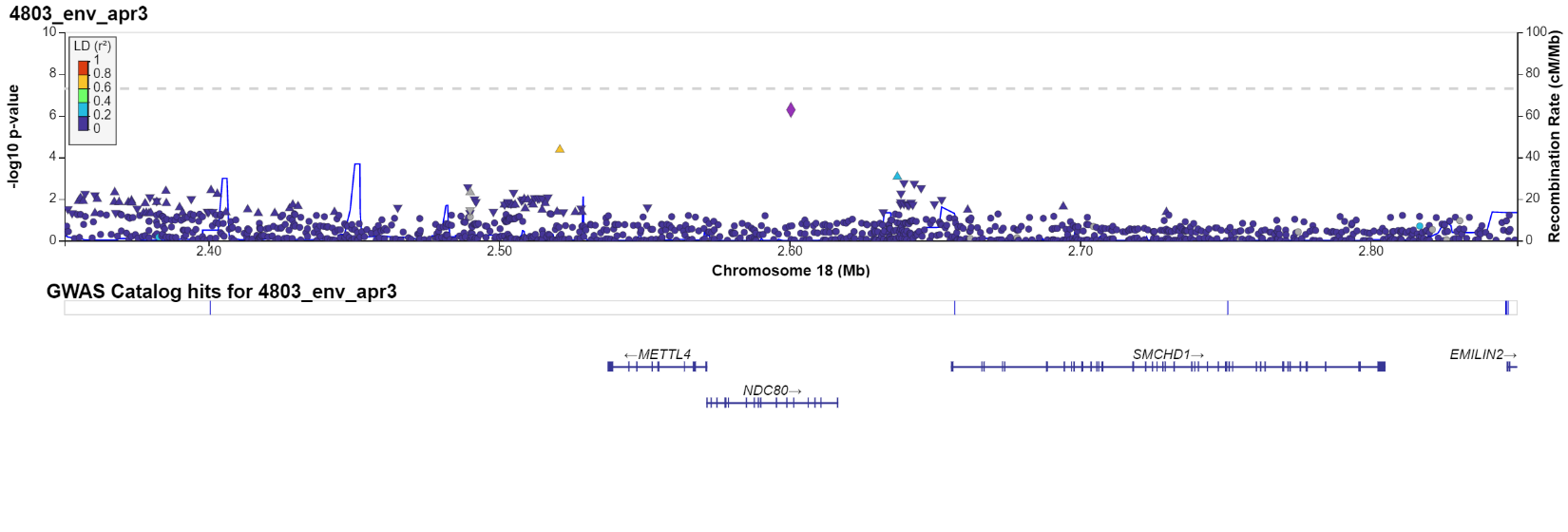


Figure 2N: Chr: position: 2: 60,073,205 (BCL11A)


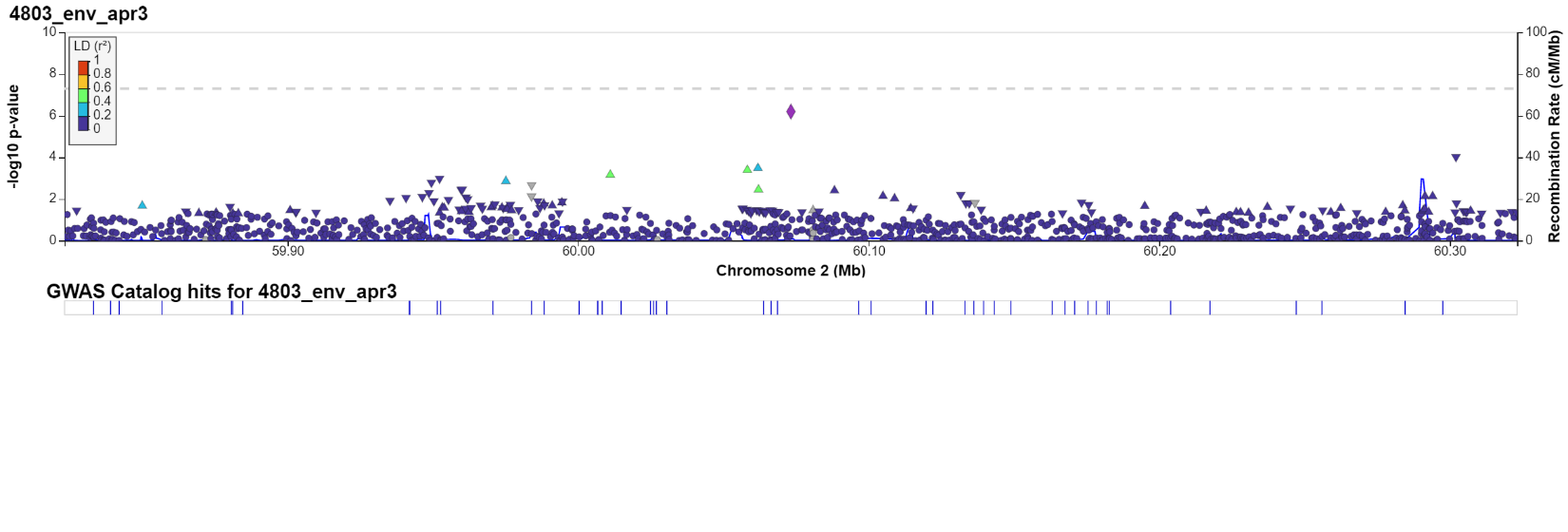


Figure1O: Chr: position: 14: 72,429,274 (RGS6)


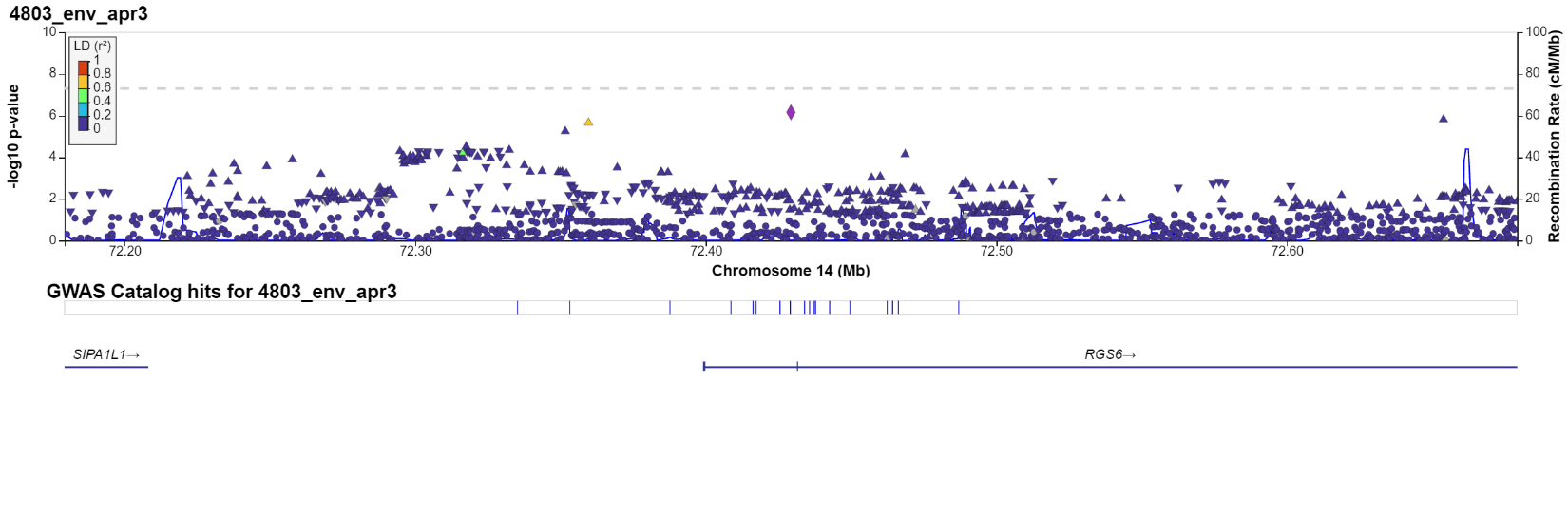


Figure 2P: Chr: position: 8: 69,546,107 (C8orf34)


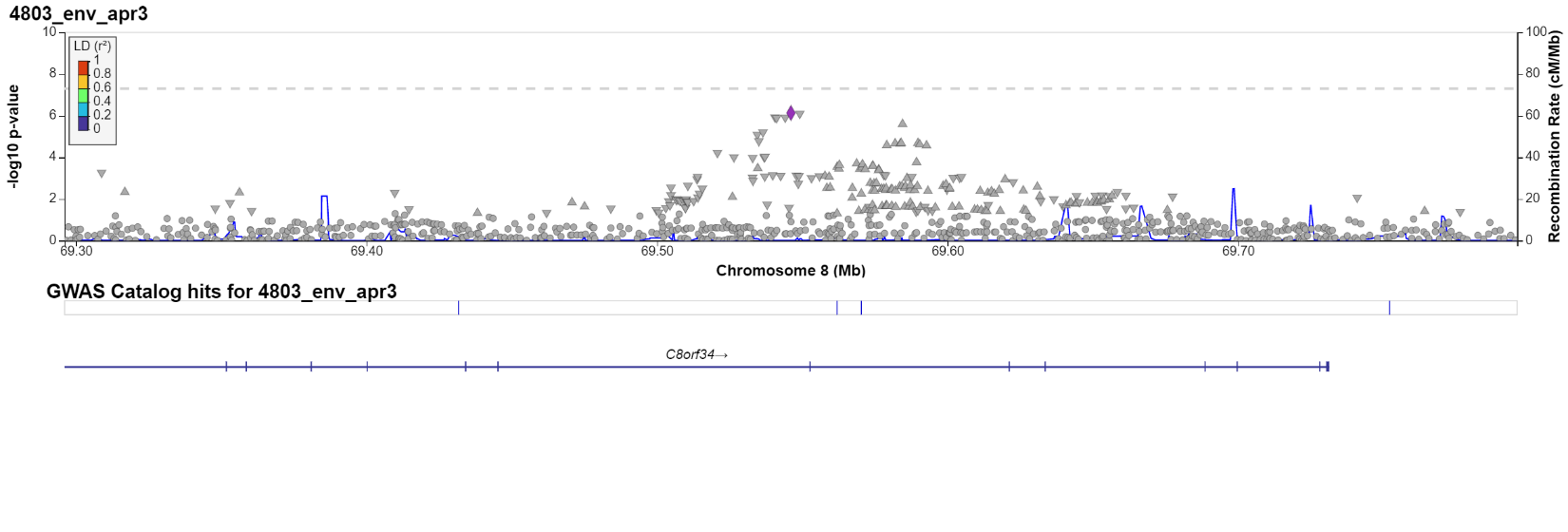


Figure 2Q: Chr: position: 7: 3,425,301 (SDK1)


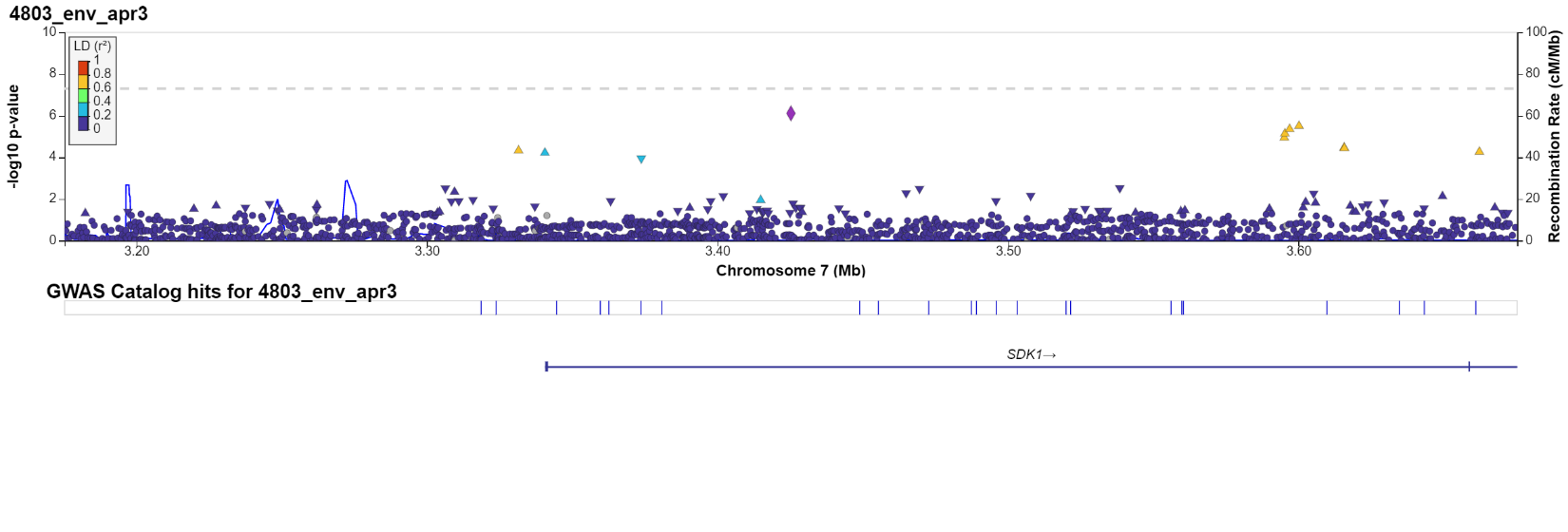


Figure 2R: Chr: position: 1: 103,448,242 (COL11A1)


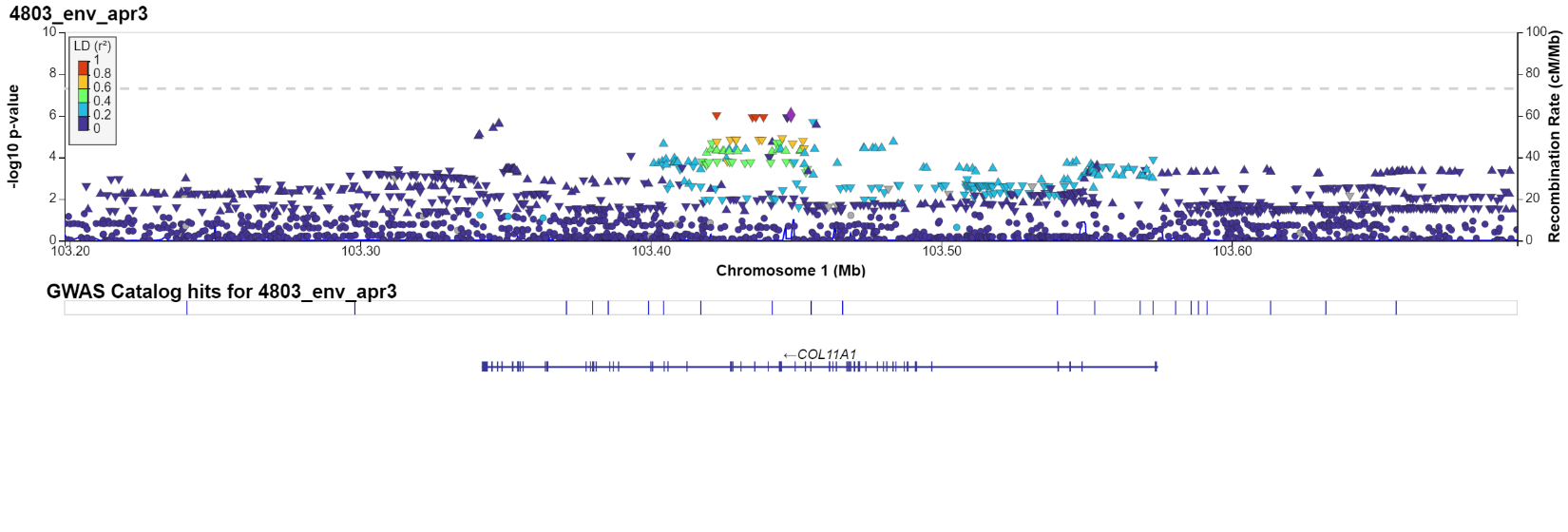


Figure 2S: Chr: position: 10: 73,582,752 (PSAP)


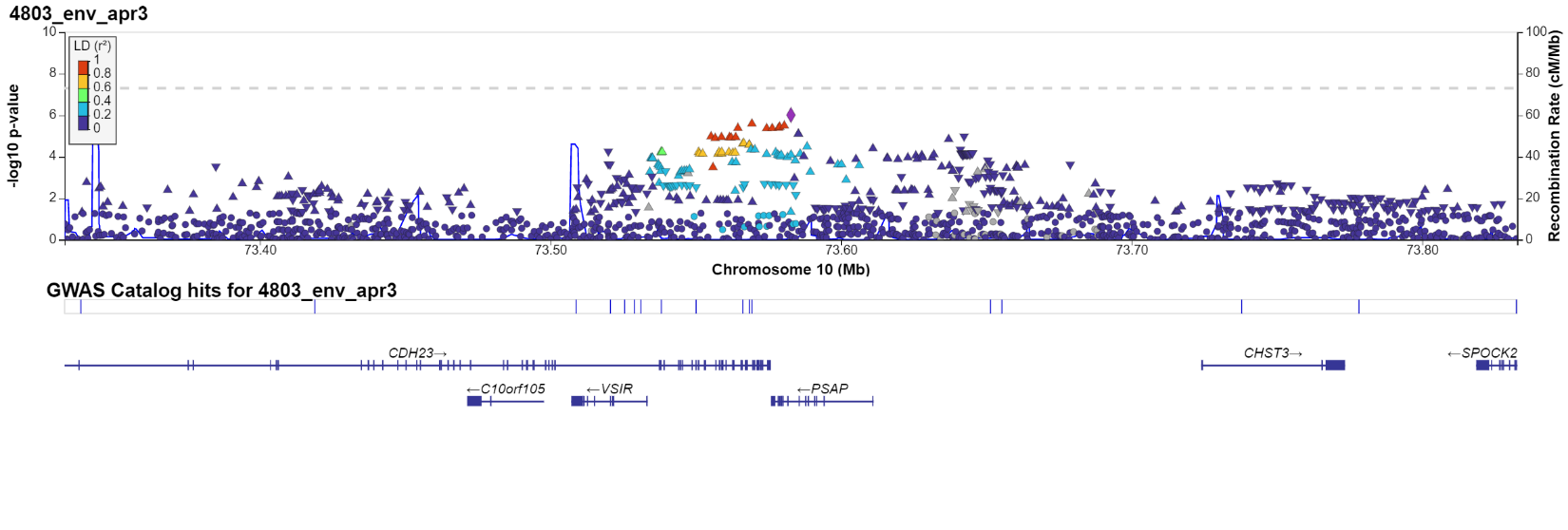


Figure 2T: Chr: position: 13: 93,040,866 (GPC5)


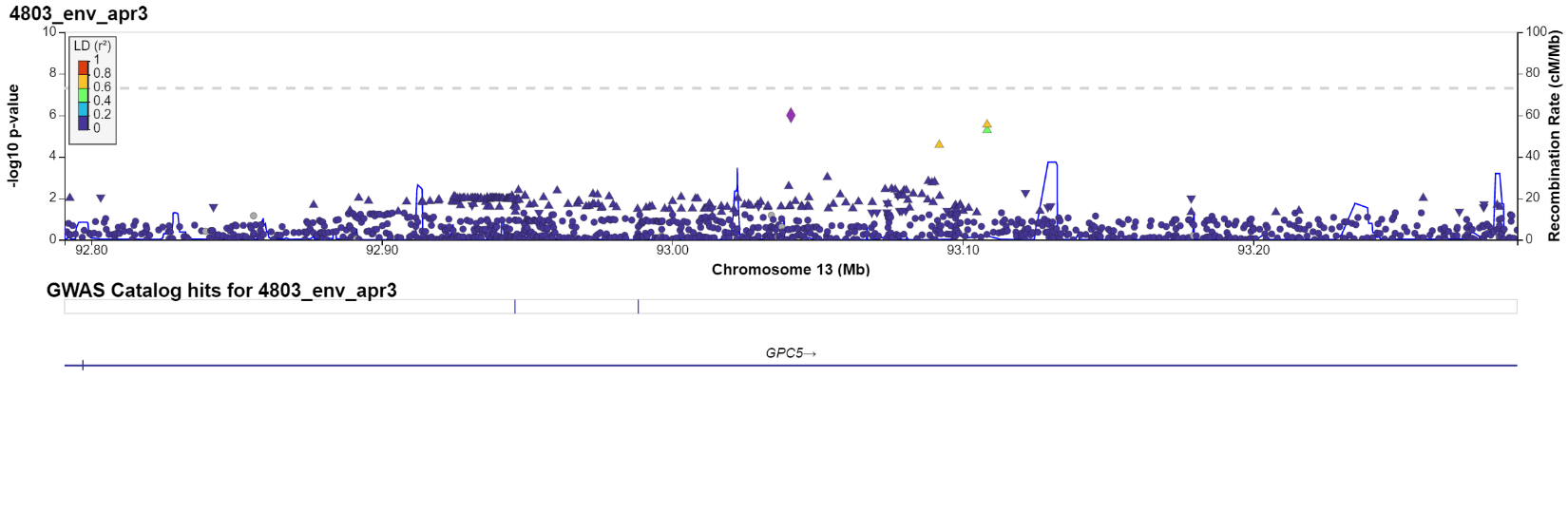


**Figure 3: Manhattan plot: Gene-based test (FUMA) - TINNITUS (Data-field: 4803)**


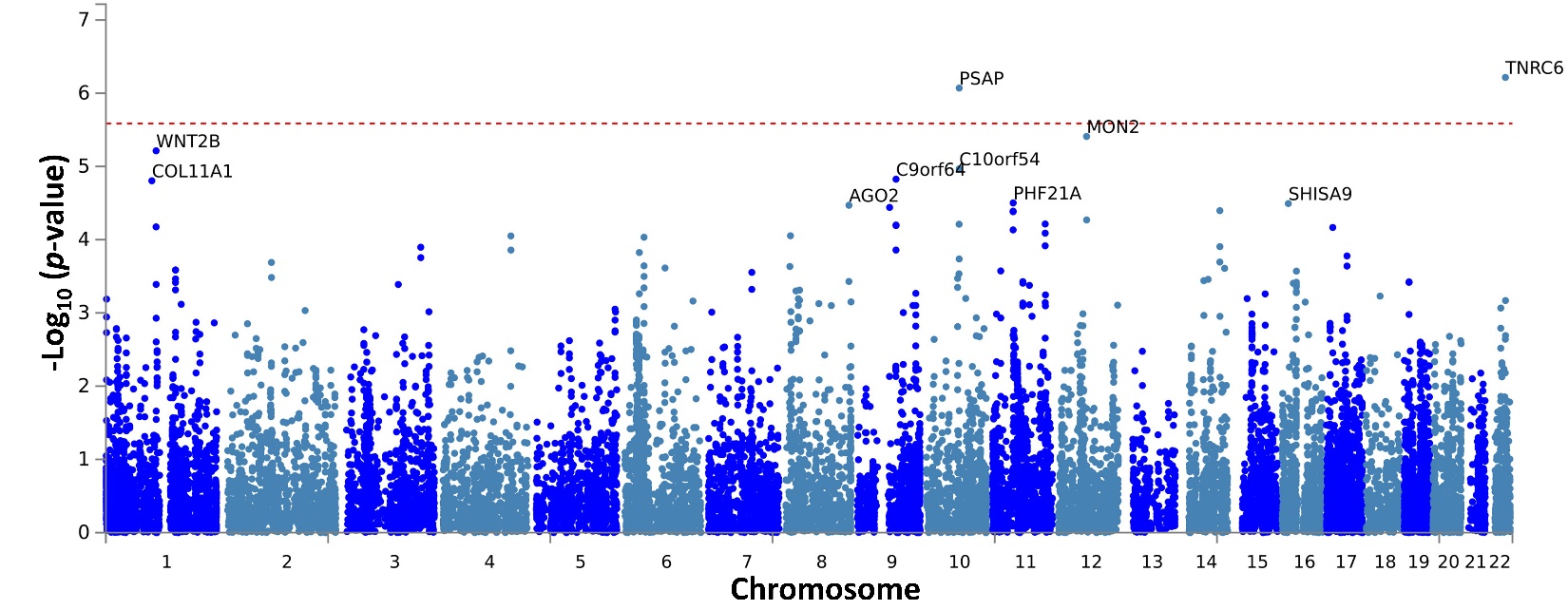


**Figure 4: Tissue specificity (GTEX_V8) analysis (FUMA) - TINNITUS (Data-field: 4803)**


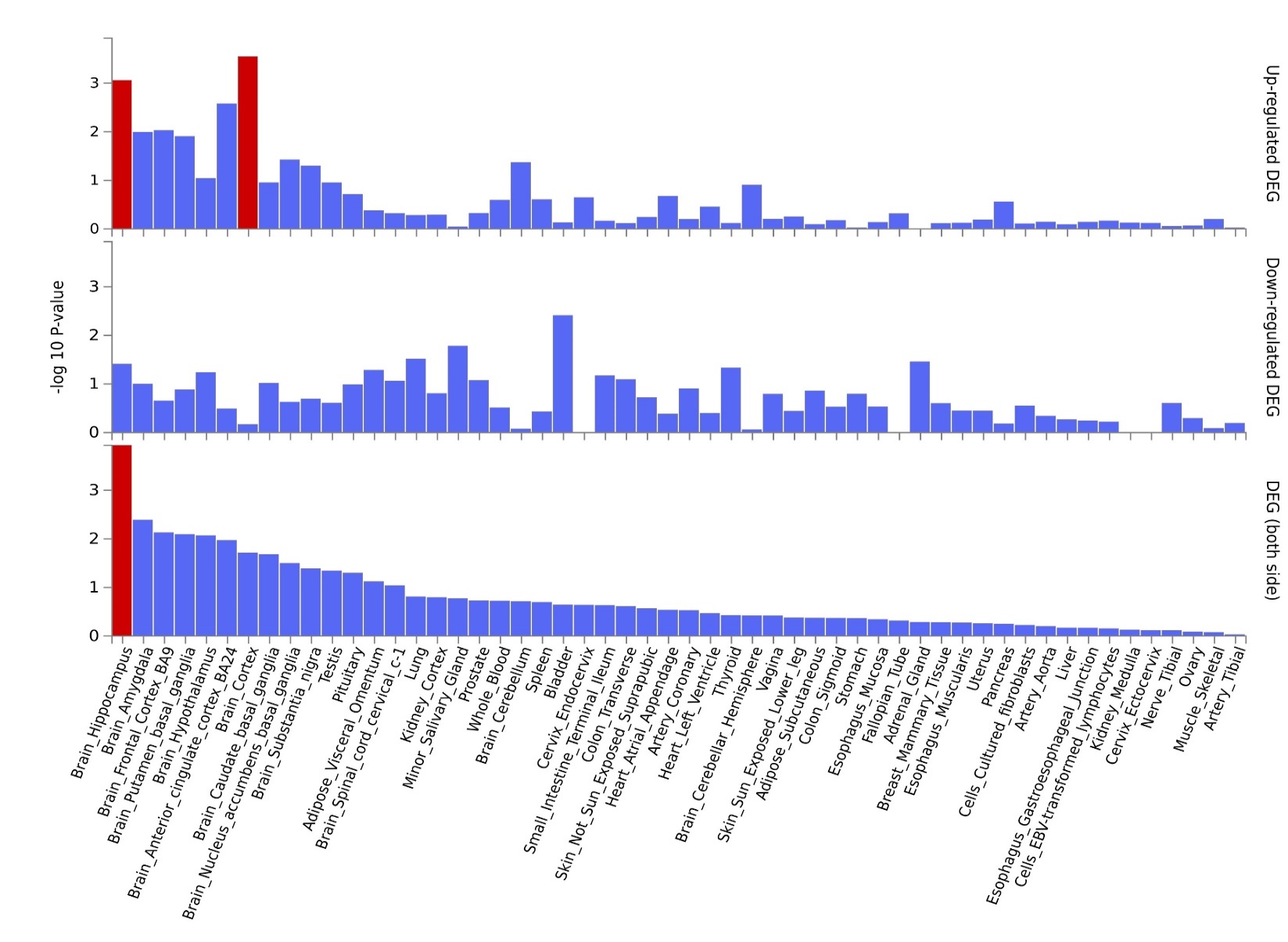


**Figure 5: Quantile-Quantile plots of expected and observed *p*-values (converted on a -log10 (*p*-value) scale) for the genome-wide association study model (TINNITUS (Data-field: 4803). The plot on the right-hand side shows Q-Q plots for MAF categories and shows genomic inflation measurement (GC lambda).**


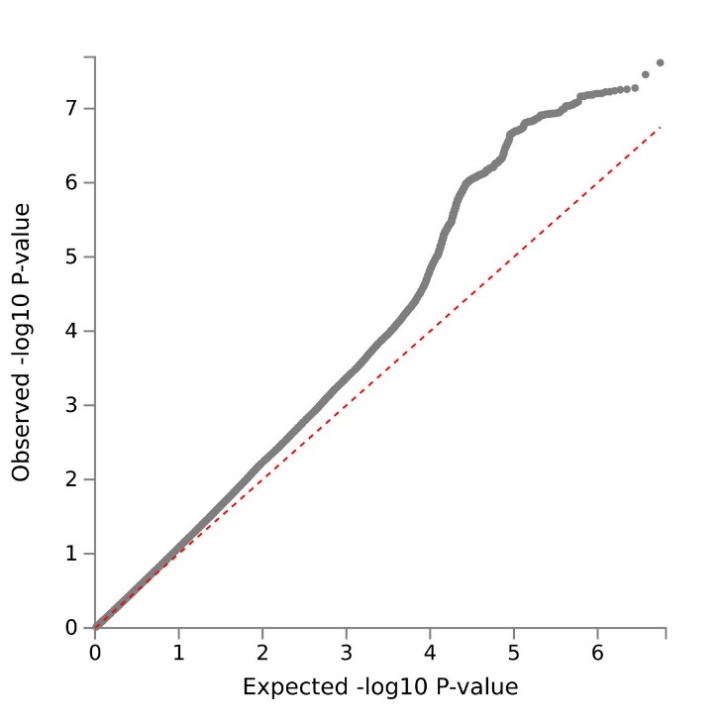

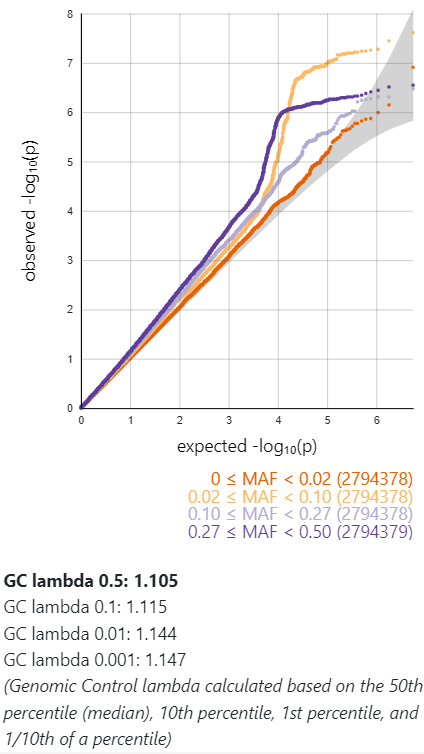


**Figure 6: Results of the enrichment analysis: Tinnitus (Data-field: 4803)**

Figure 6A: Positional gene sets (MsigDB c1)


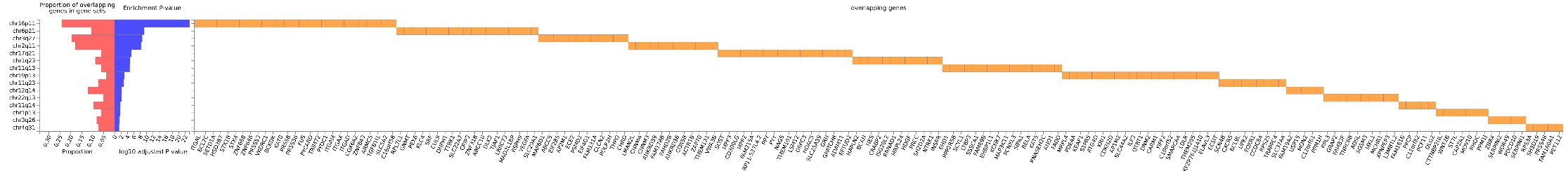


Figure 6B: TF targets (MsigDB c3)


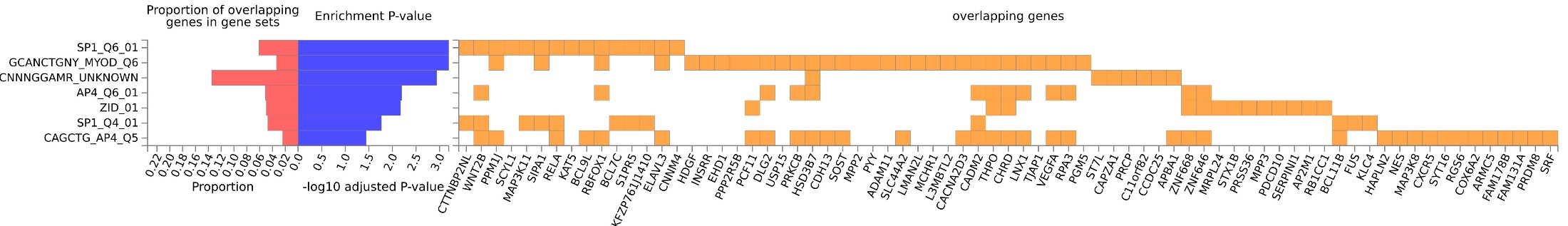


Figure 6C: GWAS catalog reported genes


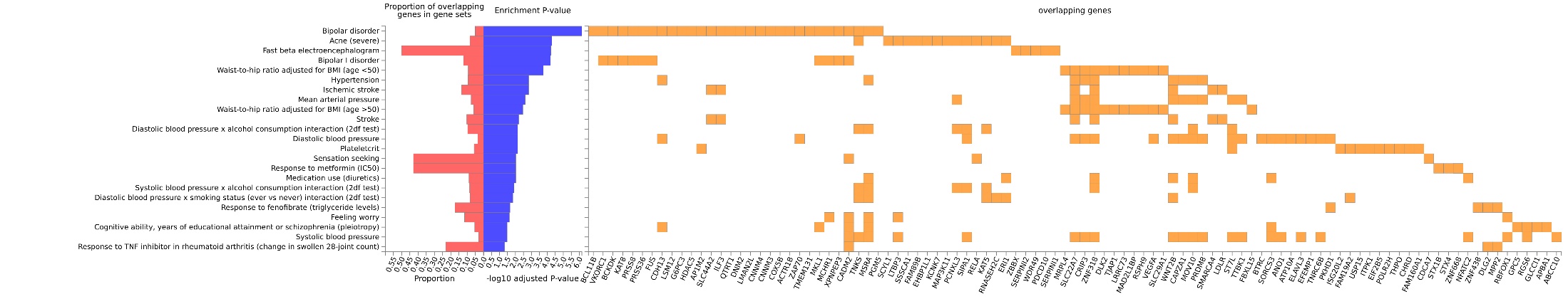


**Figure 7: LocusZoom: TINNITUS-RELATED DISTRESS (Data-field: 4814)**

Figure 7A: Chr: position: 4: 35,067,243 (snoU13)


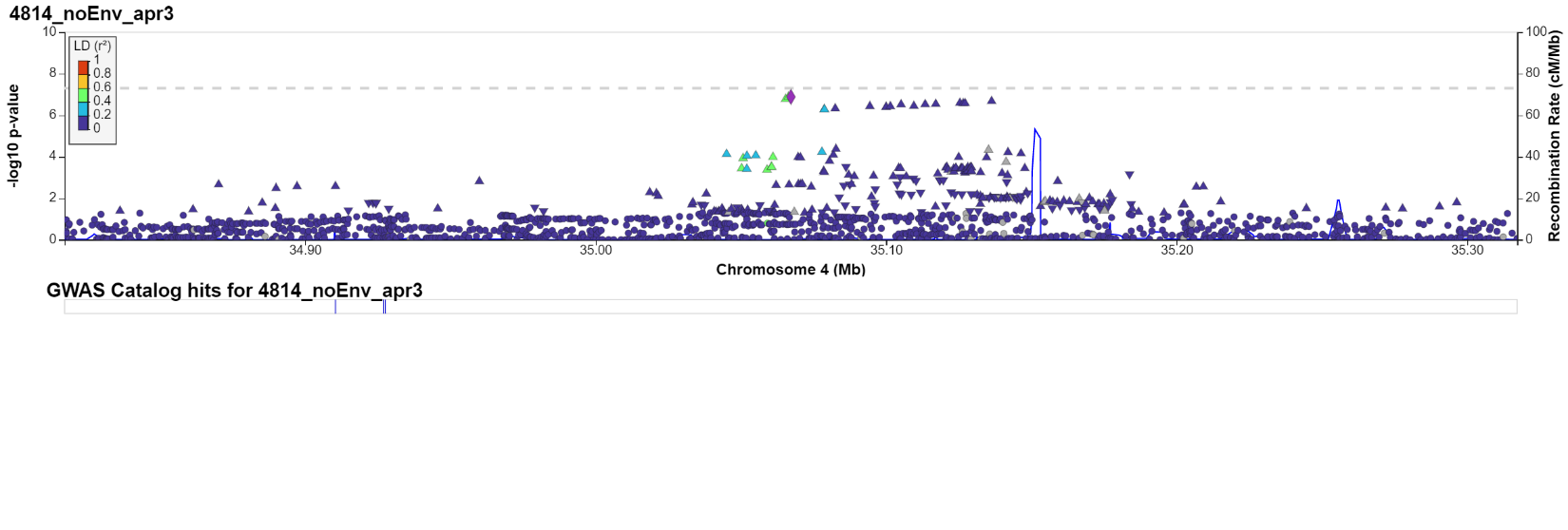


Figure 7B: Chr: position: 4: 176,291,501 (GPM6A)


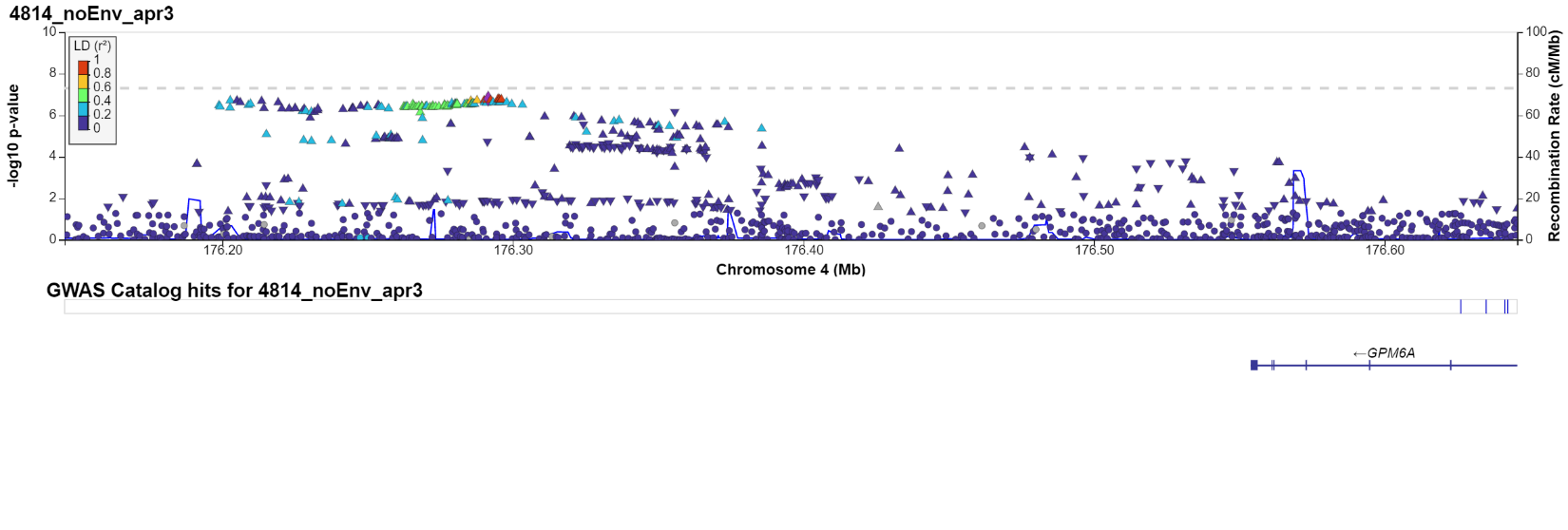


Figure 7C: Chr: position: 4: 183,705,777 (TENM3)
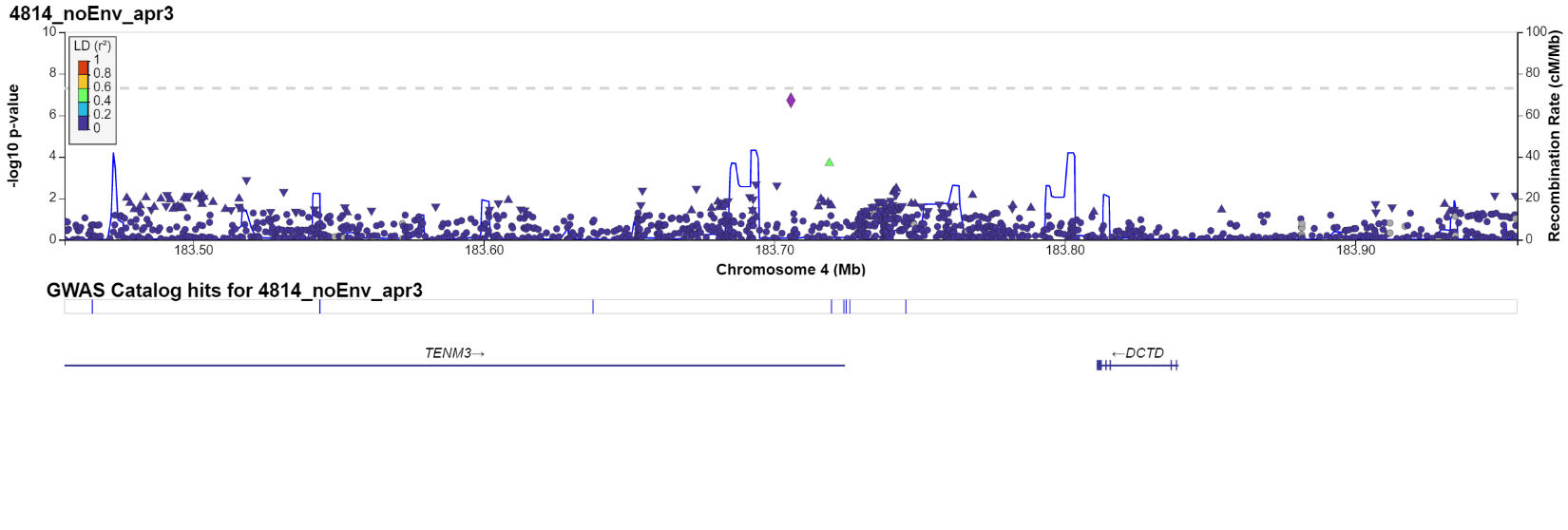


Figure 7D: Chr: position: 9: 86,553,664 (C9orf64)
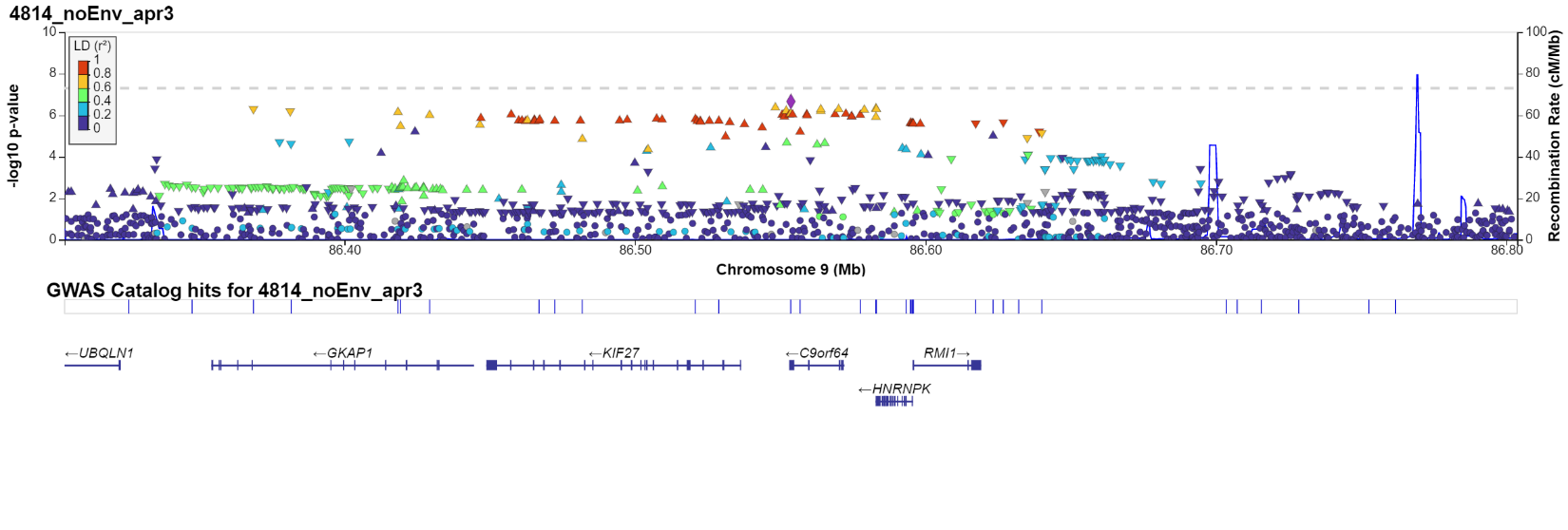


Figure 7E: Chr: position: 2: 140,770,563 (RN7SL283P/LRP1B)


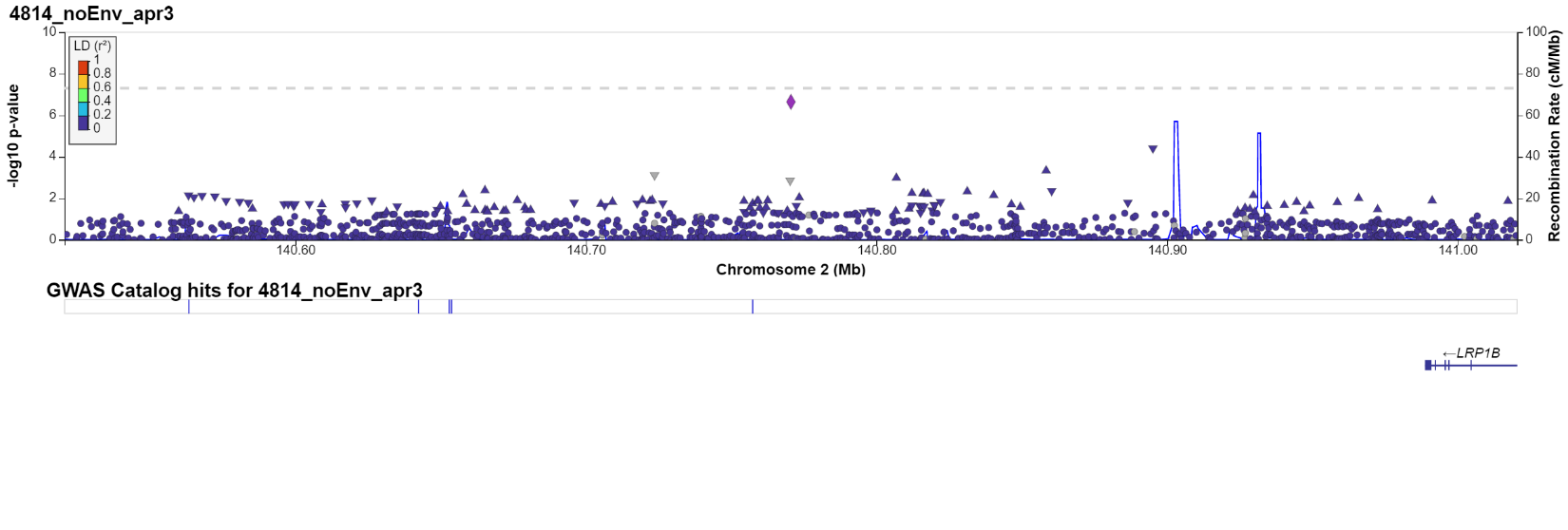


Figure 7F: Chr: position: 8: 141,576,720 (AGO2)


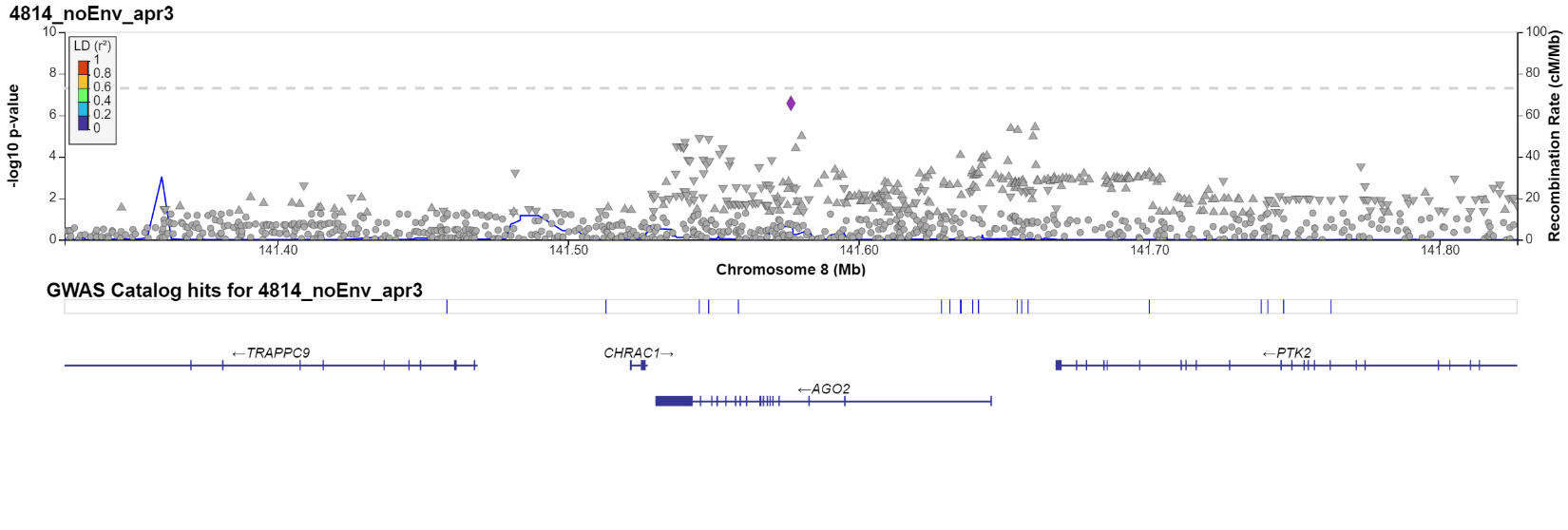


Figure 7G: Chr: position: 11: 62,596,218 (STX5)


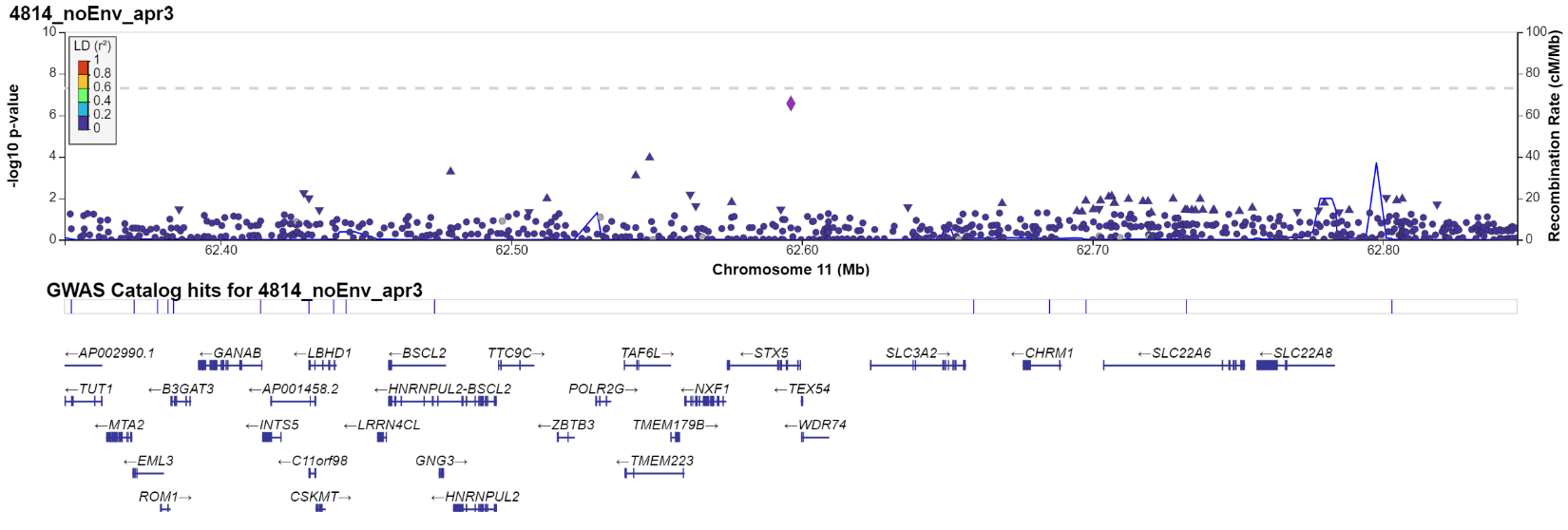


Figure 7H: Chr: position: 2: 75,699,744 (EVA1A)


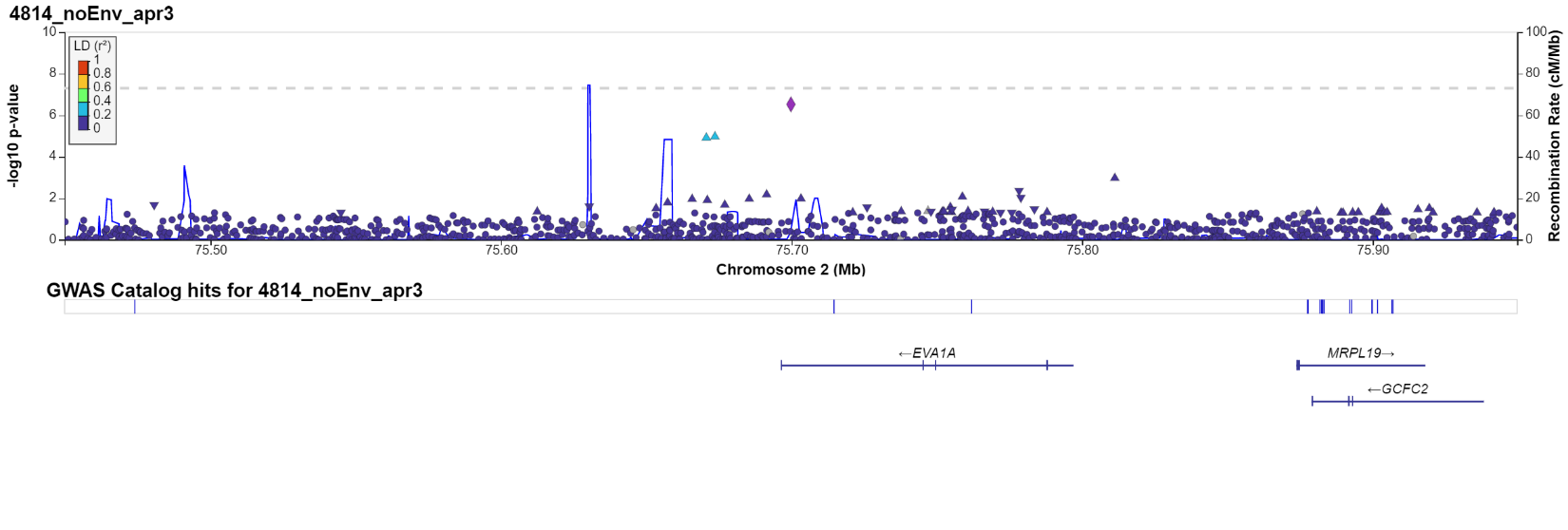


Figure 7I: Chr: position: 17: 54,624,719 (RPL39P33/ANKFN1)


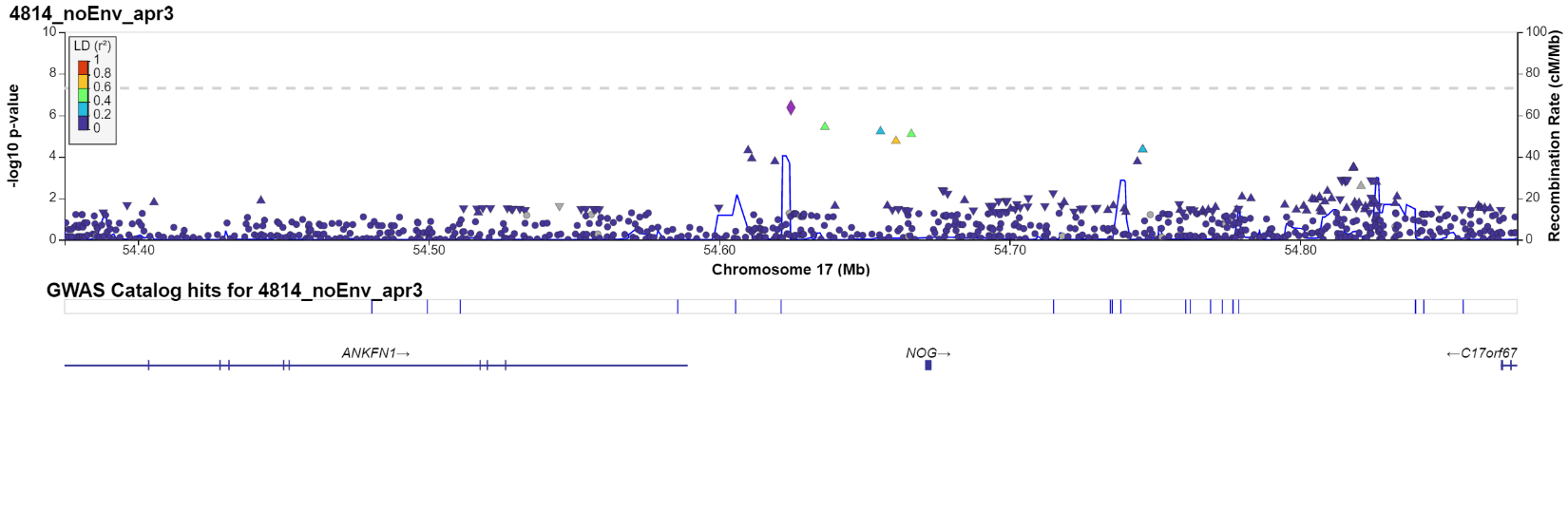


Figure 7J: Chr: position: 16: 7,646,138 (RBFOX1)


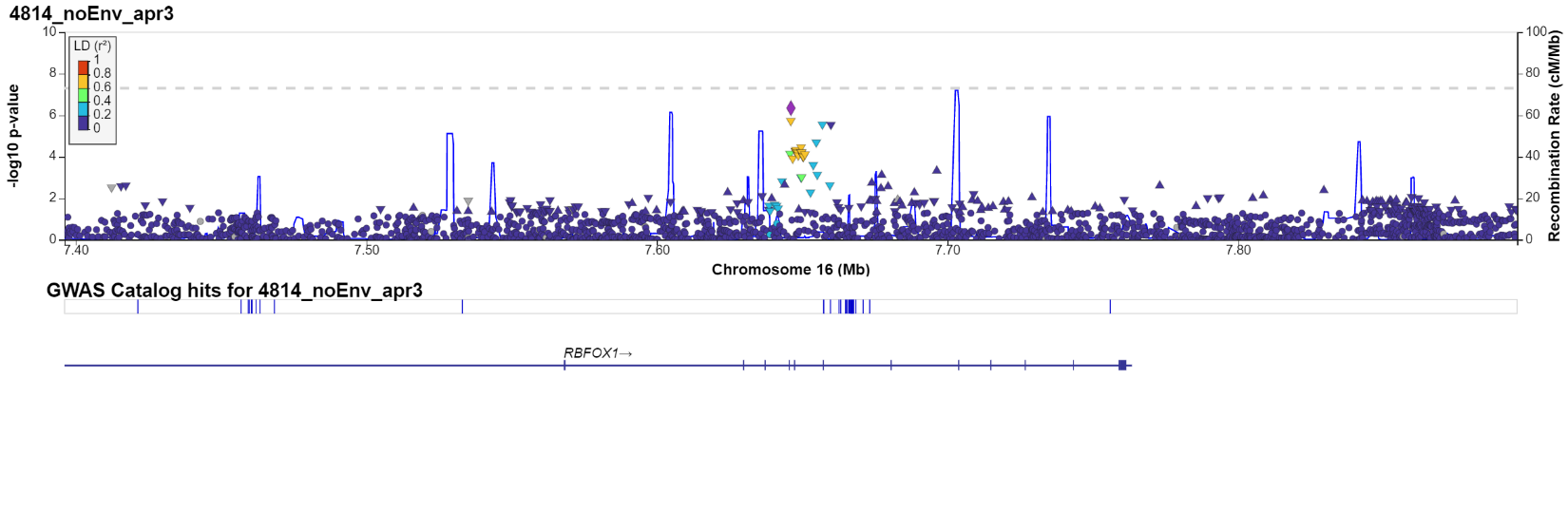


Figure 7K: Chr: position: 10: 129,362,692 (NPS)


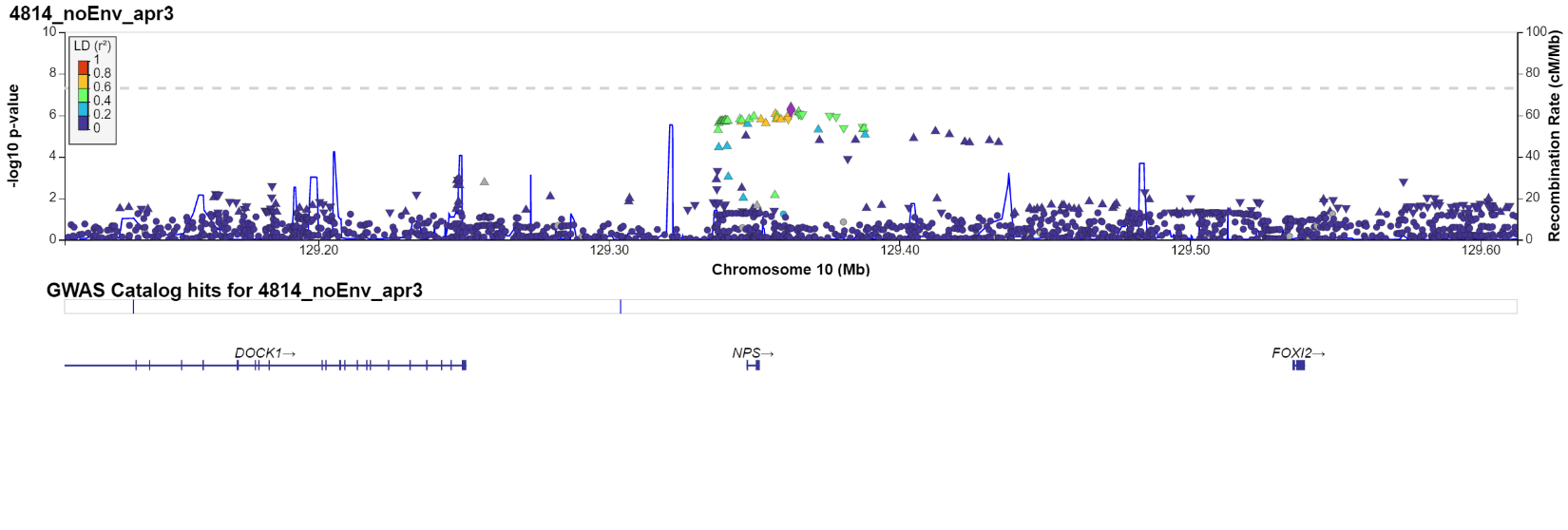


Figure 7L: Chr: position: 18: 3,436,449 (TGIF1)


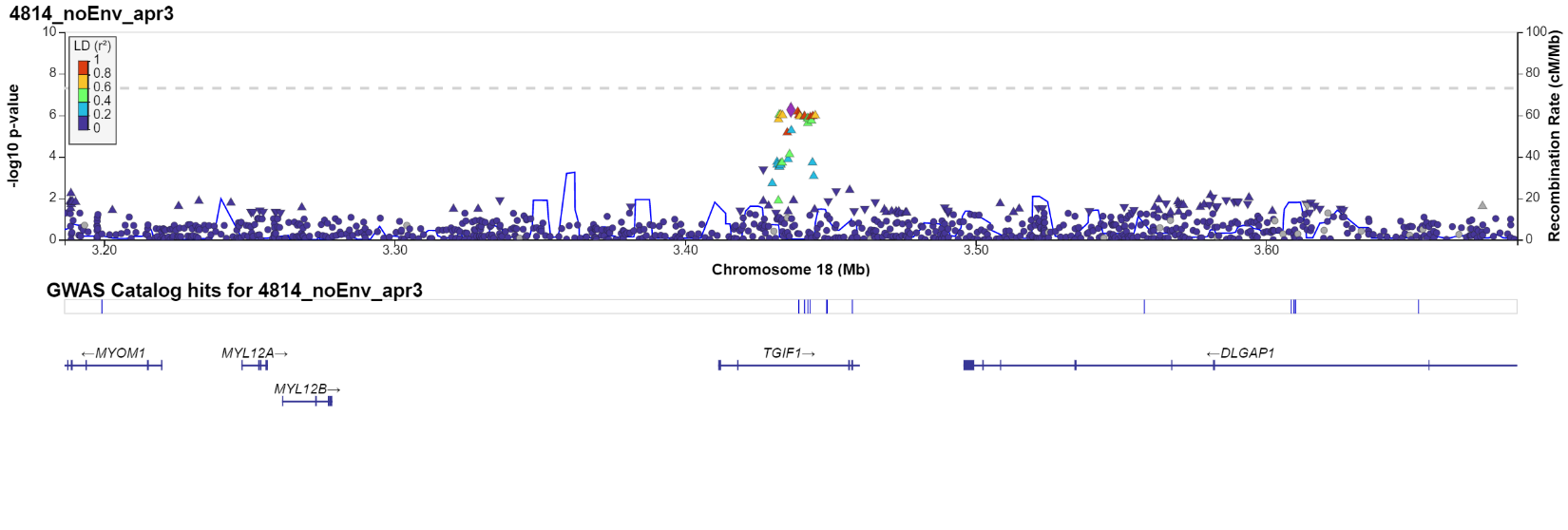


Figure 7M: Chr: position: 2: 41,551,500 (HNRNPA1P57)


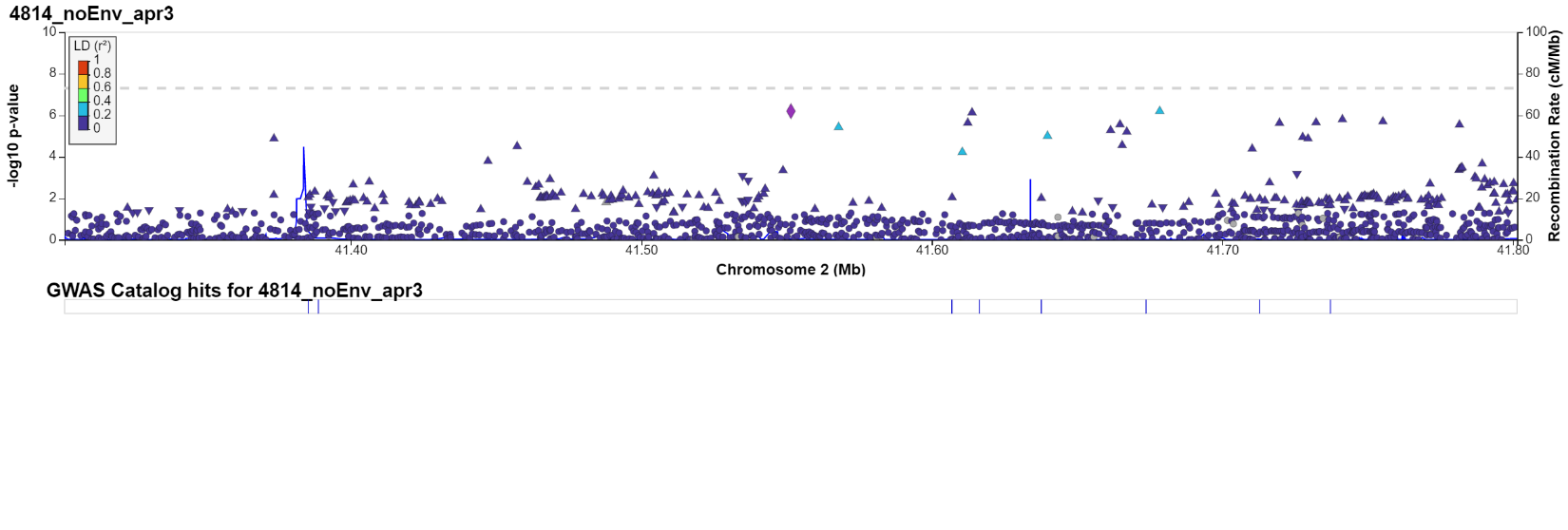


Figure 7N: Chr: position: 16: 9,174,460 (C16orf72)


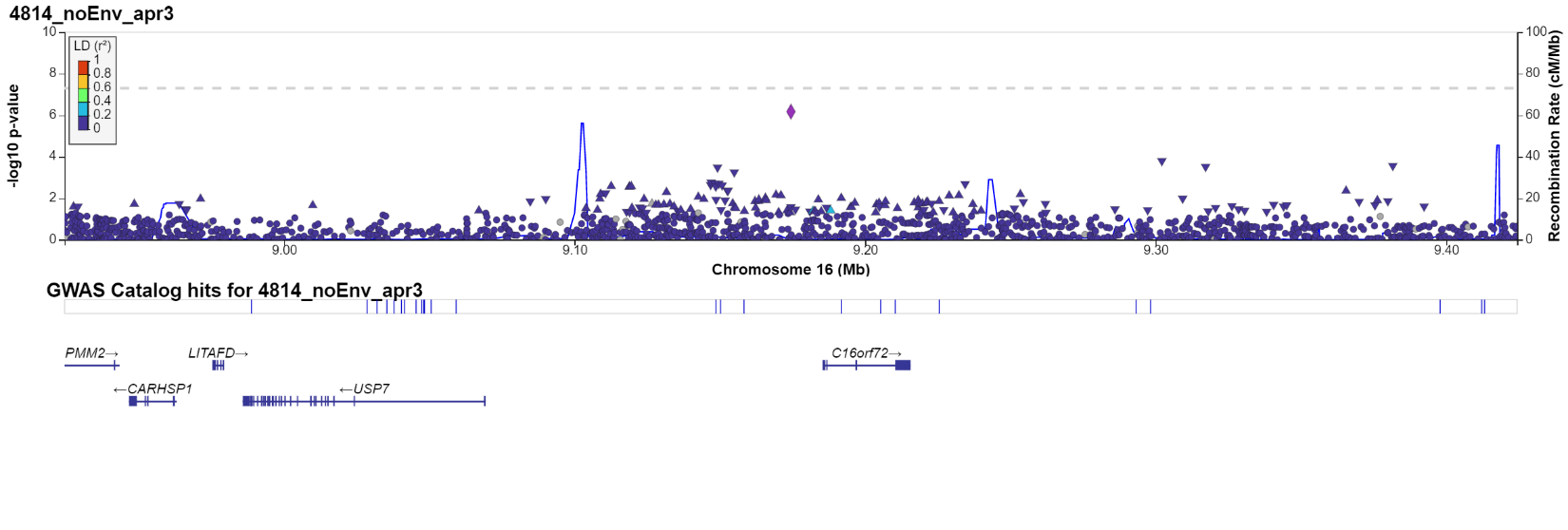


Figure 7O: Chr: position: 14: 88,972,303 (PTPN21/AL162171.2)


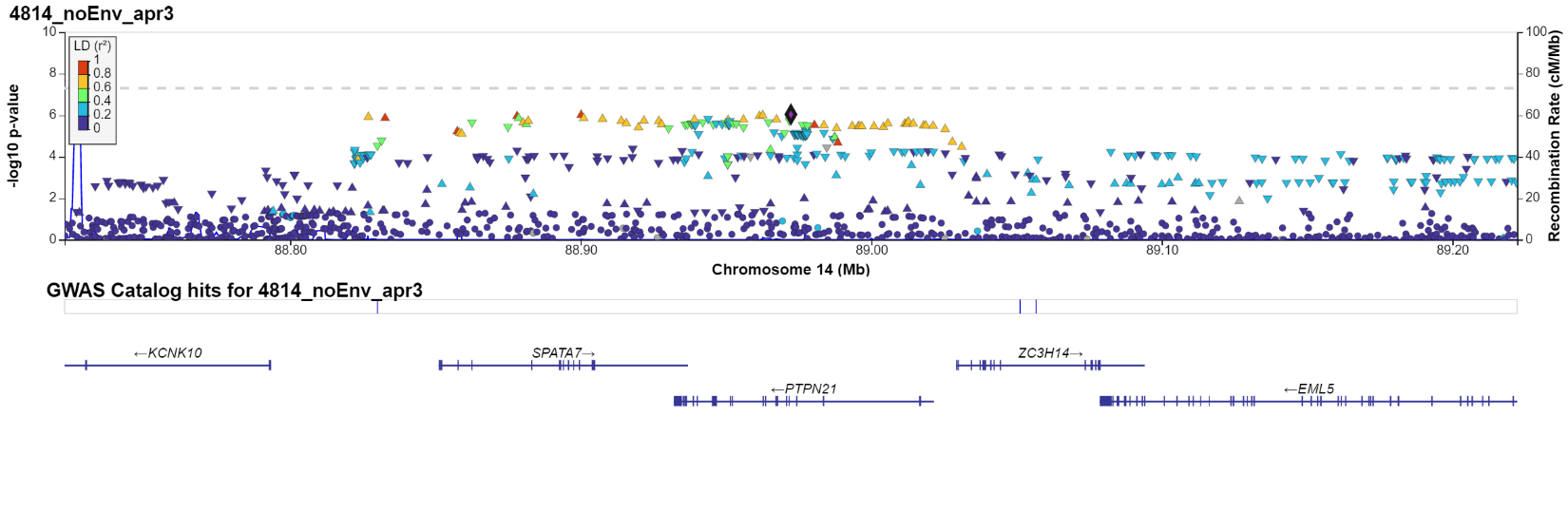


**Figure 8: Manhattan plot: Gene-based test (FUMA) - TINNITUS-RELATED DISTRESS (Data-field: 4814)
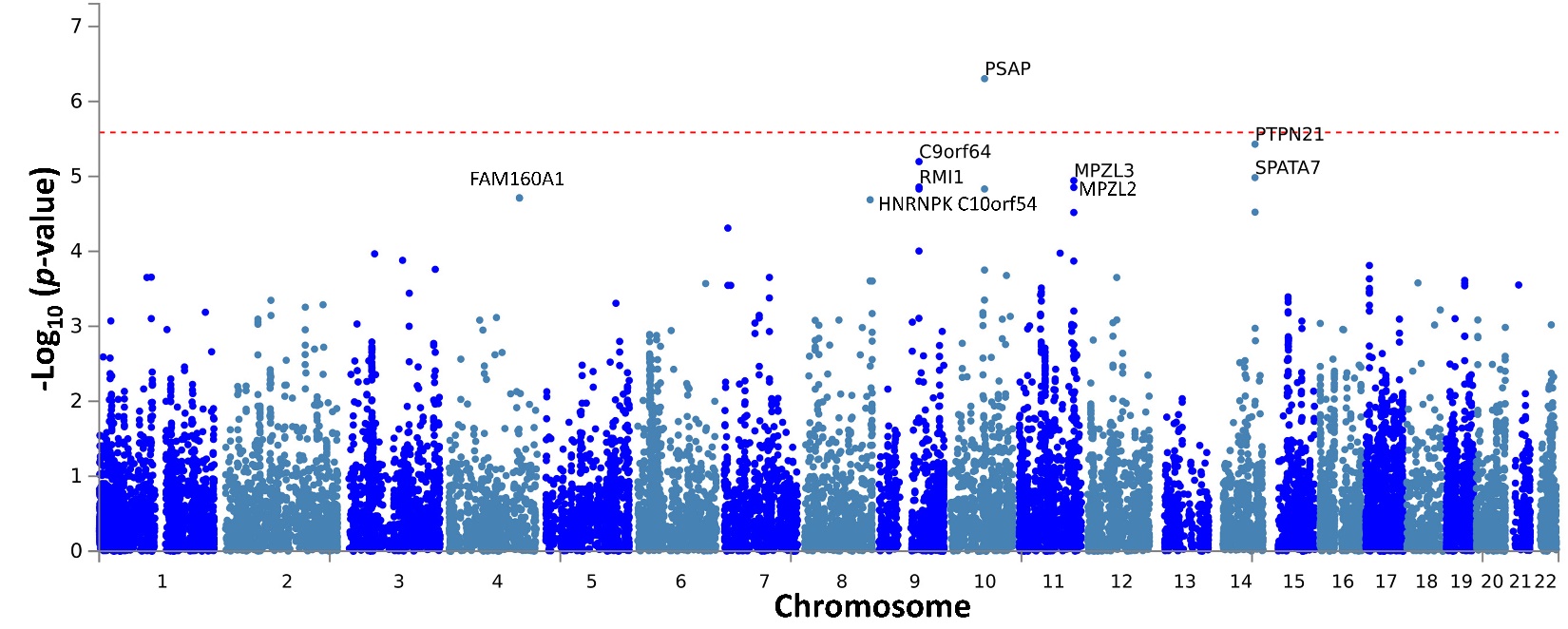
**

**Figure 9: Tissue specificity (GTEX_V8) analysis (FUMA) - TINNITUS-RELATED DISTRESS (Data-field: 4814)
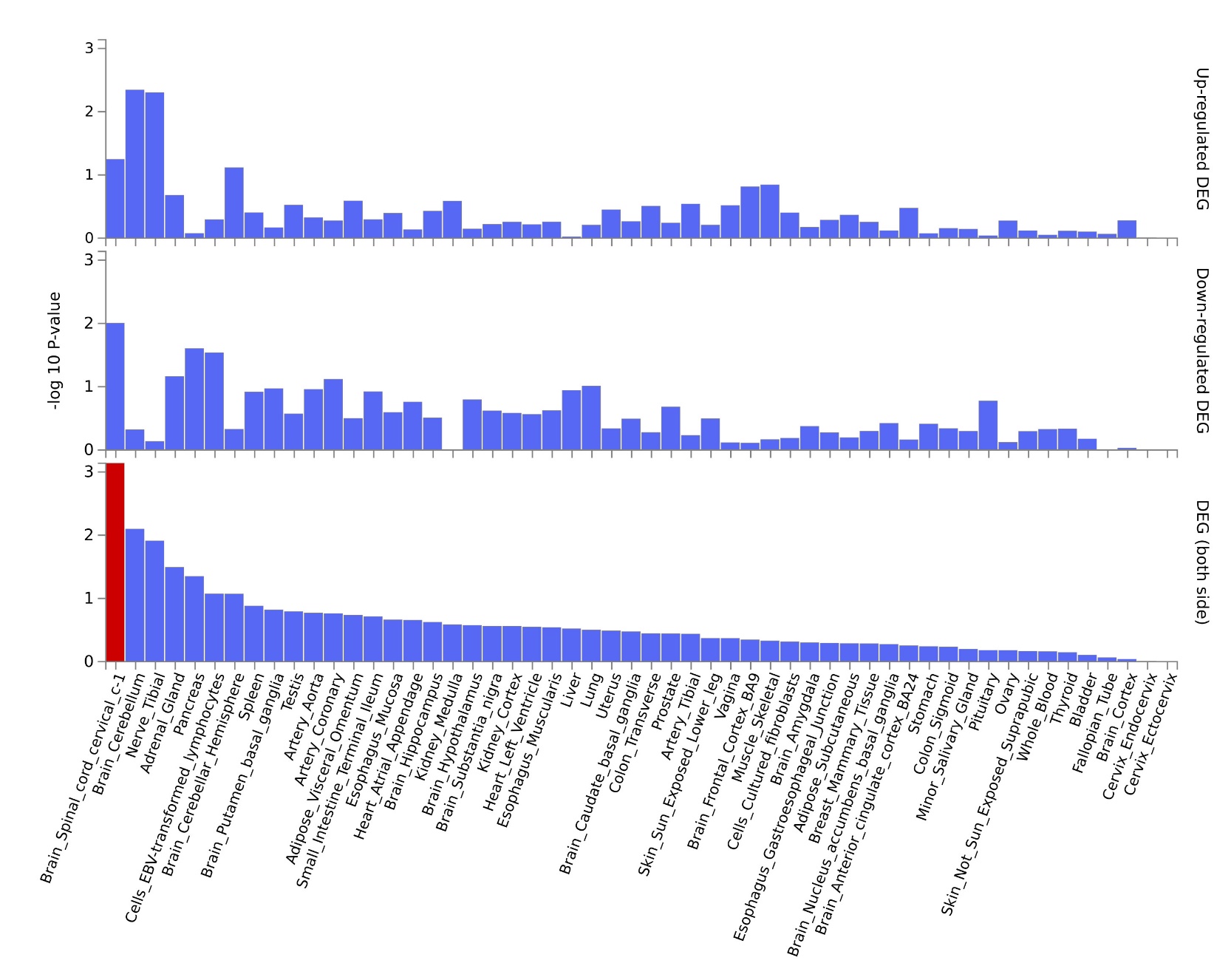
**

**Figure 10: Quantile-Quantile plots of expected and observed *p*-values (converted on a -log10 (*p*-value) scale) for the genome-wide association study model (TINNITUS-RELATED DISTRESS (Data-field: 4814)). The plot on the right-hand side shows Q-Q plots for MAF categories and shows genomic inflation measurement (GC lambda).**

**
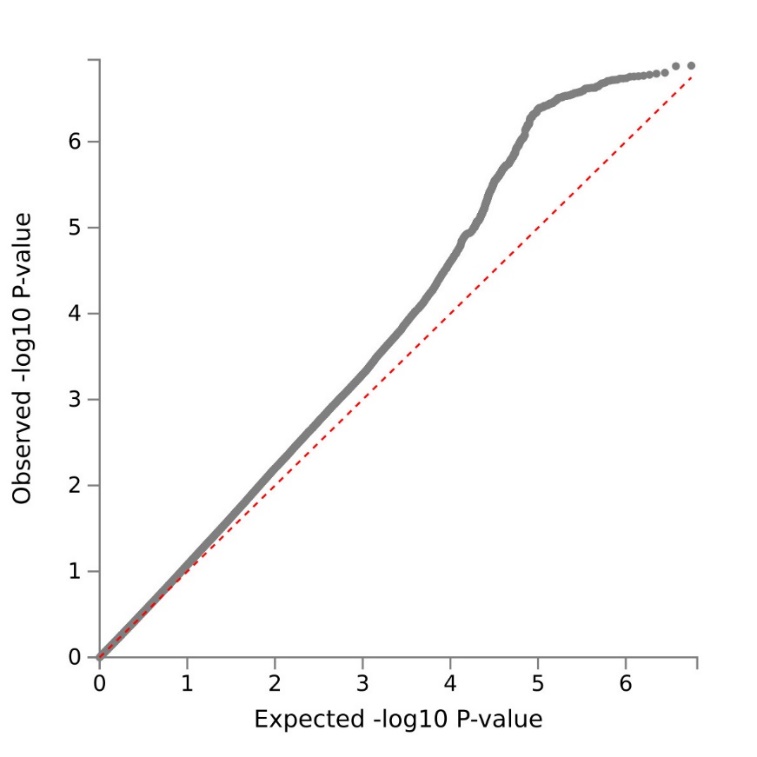
**


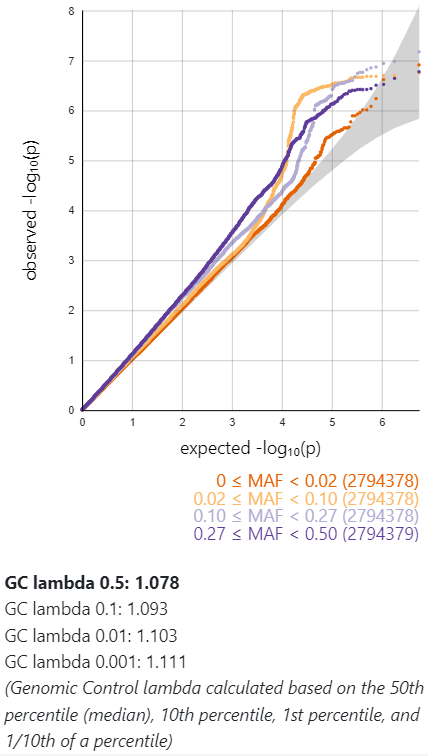


**Figure 11: Results of the enrichment analysis: Tinnitus-related distress (Data-field: 4814)**

Figure 11A: Positional gene sets (MsigDB c1)


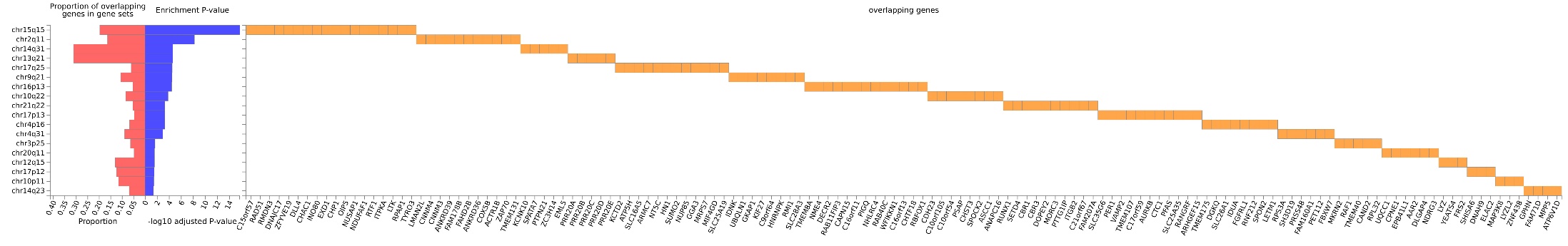


Figure 11B: Curated gene sets
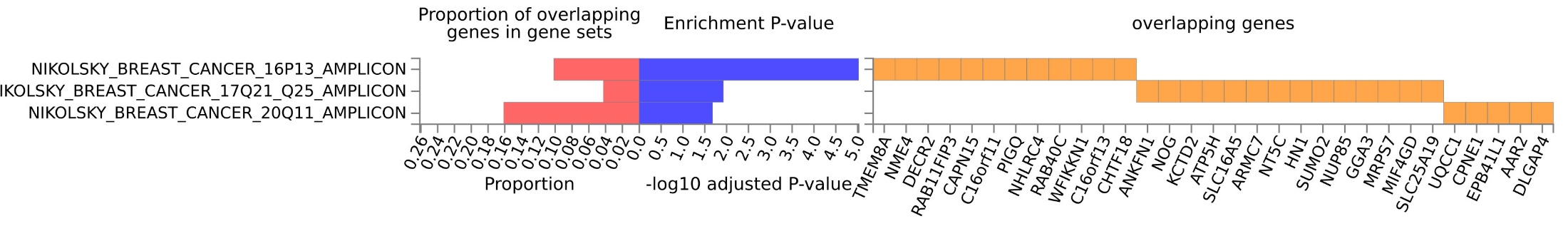


Figure 11C: Chemical and genetic perturbation gene sets (MsigDB c2)


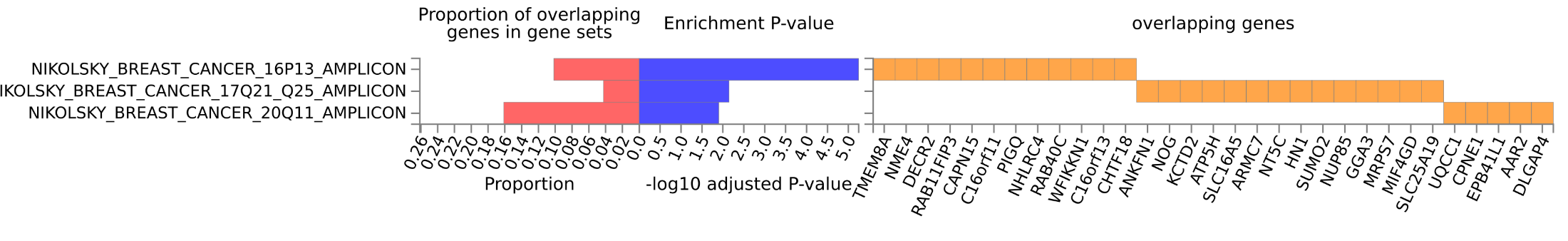


Figure 11D: GWAS catalog reported genes


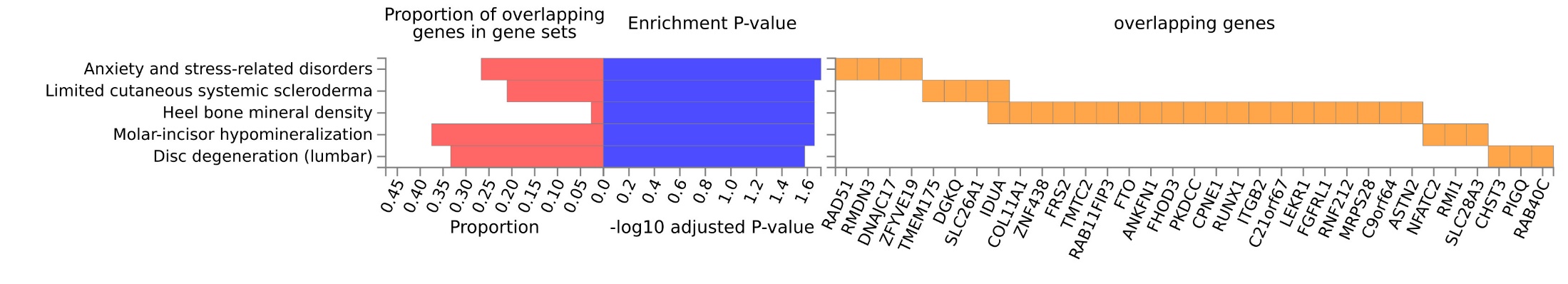


Figure 12: A scatter plot between the genomic PCA1 and PCA2 for individuals reporting tinnitus and no tinnitus. The GWAS models used 10 genomic PCAs to account for population stratification.


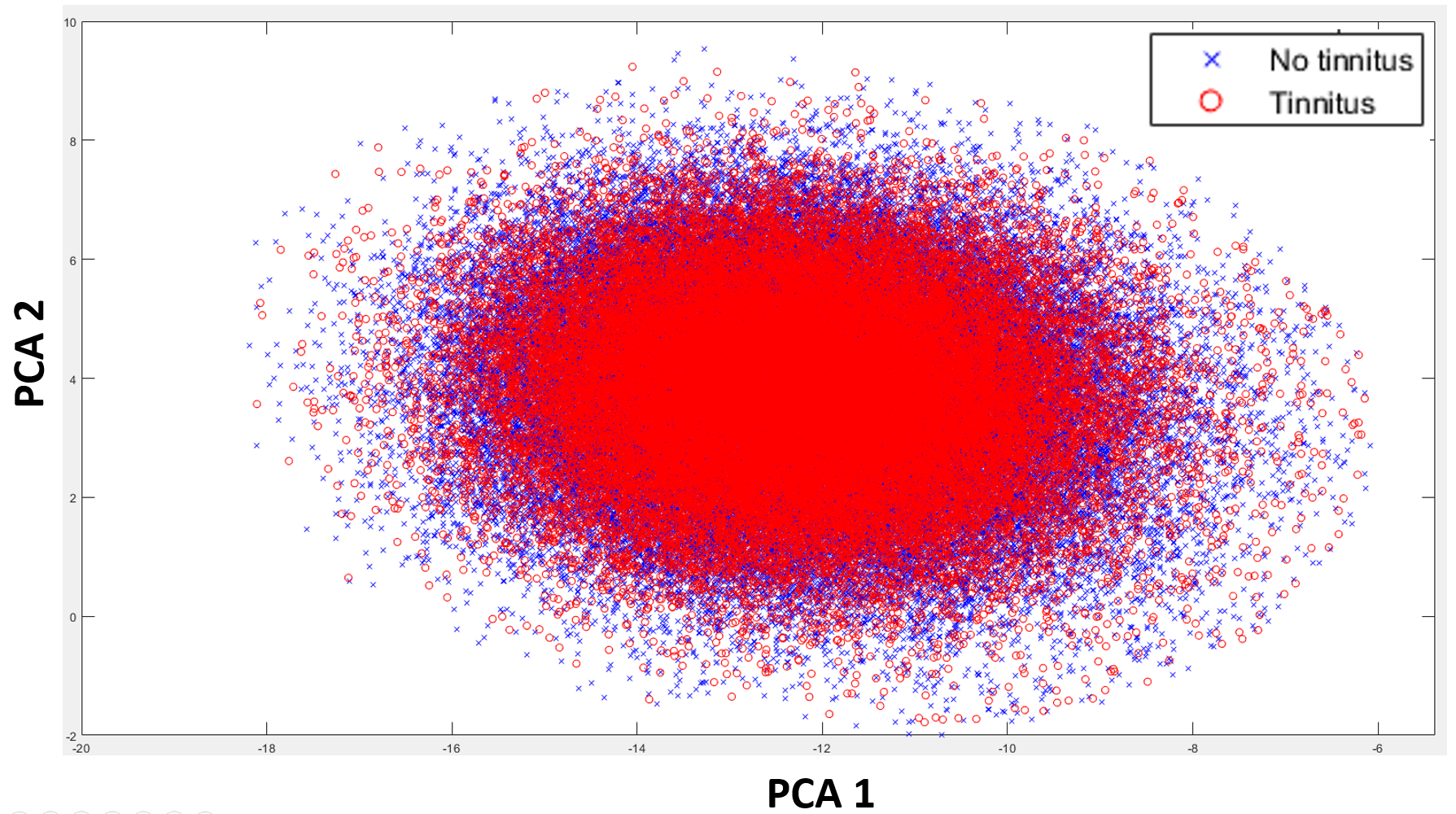


**Figure 13: GWAS power calculation.** A comparison of statistical power between the present study (N=132,438), and the sample size used by Clifford et al. (2020) (N=155,395). The present study excluded participants based on environmental covariates and ethnic background (Figure 1), resulting in a smaller sample size than Clifford et al. (2020). Our power analysis (GAS power calculator using *p*=5*10^-8^, prevalence=0.30, model: Additive) revealed that the present study had a relatively lower power for detecting small sample sizes (OR<1.15) than Clifford et al. (2020), which might contribute to the differences in the GWAS results (see Table 5).


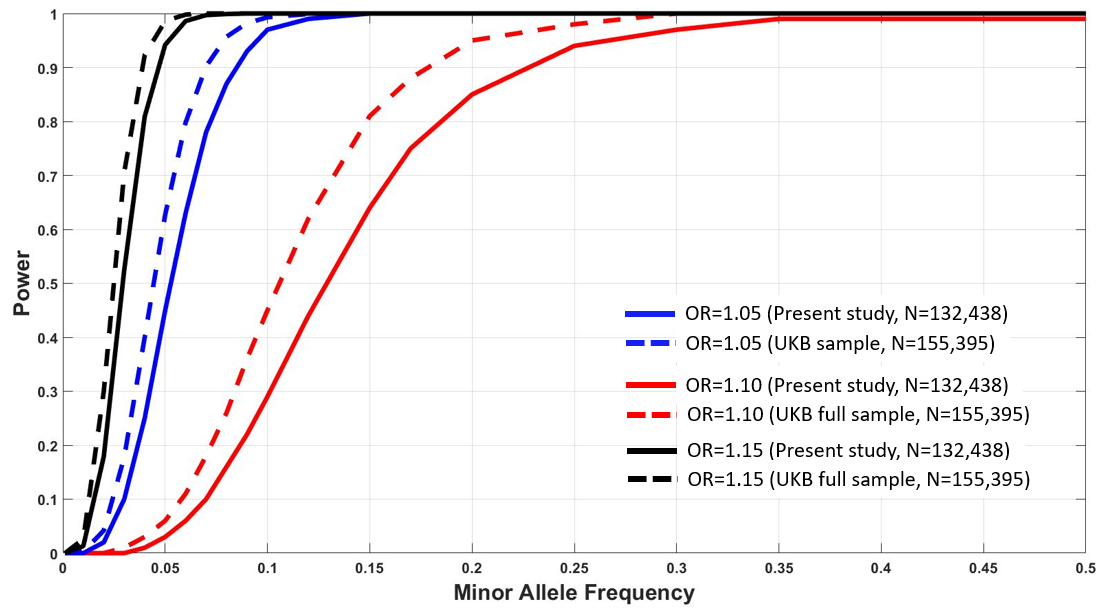


**Note:** Wells et al (2021) utilized a sample size of 91424 for the GWAS by including those with no tinnitus as controls and those reporting “Yes, now most of the time” or “Yes, now a lot of the time” as cases. We could not perform a comparable power analysis as selecting extreme cases might influence statistical power based on the phenotypic distance between cases and control.

**Reference:**

Skol, A. D., Scott, L. J., Abecasis, G. R., & Boehnke, M. (2006). Joint analysis is more efficient than replication-based analysis for two-stage genome-wide association studies. Nature genetics, 38(2), 209-213.

Clifford, R. E., Maihofer, A. X., Stein, M. B., Ryan, A. F., & Nievergelt, C. M. (2020). Novel risk loci in tinnitus and causal inference with neuropsychiatric disorders among adults of European ancestry. JAMA Otolaryngology–Head & Neck Surgery, 146(11), 1015-1025.

Wells, H. R., Abidin, F. N. Z., Freidin, M. B., Williams, F. M., & Dawson, S. J. (2021). Genome-wide association study suggests that variation at the RCOR1 locus is associated with tinnitus in UK Biobank. Scientific reports, 11(1), 1-10.
